# Cross-disease transcriptomic analysis reveals DOK3 and PAPOLA as therapeutic targets for neuroinflammatory and tumorigenic processes

**DOI:** 10.3389/fimmu.2024.1504629

**Published:** 2024-12-12

**Authors:** Xingqiao Wang, Yusong Bian, Weiguang Chen

**Affiliations:** Emergency Department, Affiliated Yantai Yuhuangding Hospital of Qingdao University, Yantai, Shandong, China

**Keywords:** pan-cancer, differentially expressed genes, biomarkers, machine learning, DOK3, PAPOLA, inflammation, oxidative stress

## Abstract

**Objective:**

Subarachnoid hemorrhage (SAH) and tumorigenesis share numerous biological complexities; nevertheless, the specific gene expression profiles and underlying mechanisms remain poorly understood. This study aims to identify differentially expressed genes (DEGs) that could serve as biomarkers for diagnosis and prognosis.

**Methods:**

Gene expression datasets (GSE122063, GSE13353, GSE161870) were analyzed using machine learning algorithms and logistic regression to identify DEGs associated with both SAH and tumorigenesis. Lasso regression and receiver operating characteristic (ROC) curve analysis were employed to evaluate the classification accuracy of these genes. Validation of critical DEGs was performed through pan-cancer analysis and experimental studies, focusing on the role of DOK3 in modulating inflammation and oxidative stress in U251MG glioblastoma and BV2 microglia cells.

**Results:**

Fifteen common DEGs were identified, with DOK3 and PAPOLA highlighted as crucial genes implicated in SAH and neurodegenerative processes. Experimental validation demonstrated that DOK3 overexpression significantly reduced pro-inflammatory cytokine levels and oxidative stress markers while enhancing antioxidant enzyme activity. Additionally, DOK3 influenced tumorigenic processes such as apoptosis, cell cycle regulation, and proliferation, effectively mitigating LPS-induced cytotoxicity and inflammation in BV2 microglial cells.

**Conclusions:**

DOK3 and PAPOLA play critical roles in both SAH and related neurodegeneration, presenting themselves as potential prognostic biomarkers and therapeutic targets. Notably, DOK3 exhibits potential as an antitumor agent with anti-inflammatory and antioxidative properties, offering therapeutic benefits for both cancer and neuroinflammatory conditions.

## Introduction

Aneurysmal subarachnoid hemorrhage (aSAH) is a critical cerebrovascular condition characterized by the rupture of an intracranial aneurysm, leading to blood leakage into the subarachnoid space (1, 2). Among older individuals, traumatic dissection contributes to approximately 5% of all strokes, with an incidence rate of around 9 per 100,000 (3). Despite significant advancements in treatment and monitoring strategies, the mortality rate for aSAH remains approximately 40% (4, 5). Furthermore, around 40% of aSAH survivors experience persistent neurological deficits (6). Dementia, marked by memory impairment and cognitive decline, impacts daily activities, learning, work, and social engagement (7, 8). Cerebrovascular mechanisms significantly contribute to its onset, with vascular dementia accounting for 20%-40% of all dementia cases, making it the second most prevalent form after Alzheimer’s disease (9, 10).

The primary objective of this study is to explore the potential molecular links between subarachnoid hemorrhage (SAH) and vascular dementia (VaD) using integrative bioinformatics approaches, including the integration of multiple datasets (11, 12). Network analysis of miRNA-mRNA interactions provides a comprehensive bioinformatics framework for uncovering complex relationships between miRNAs and their target mRNAs (13, 14). By identifying shared differentially expressed genes (DEGs) and constructing miRNA-mRNA interaction networks, we aim to elucidate the interconnections among tumors, dementia, and cerebral hemorrhage, and to identify novel therapeutic targets that could benefit patients with SAH, VaD, and even cancer (15, 16). Transcriptomic analysis plays a crucial role in identifying DEGs across various disease states, thereby providing valuable biomarkers for diagnosis and prognosis (17–19). This approach is particularly vital in investigating complex biological processes such as SAH and tumorigenesis, which share overlapping biological complexities. Despite these shared complexities, the specific gene expression profiles and underlying mechanisms of SAH and tumor development remain largely unexplored. Consequently, identifying DEGs associated with these conditions may reveal potential biomarkers for diagnosis and prognosis, paving the way for evidence-based, personalized treatment strategies.

The combination of big data and bioinformatics allows researchers to integrate transcriptomic and proteomic data across a broad spectrum of diseases to identify potential therapeutic targets. Such multi-level data integration and analysis is extensively applied in research on cancer, neurodegenerative disorders like Alzheimer’s disease, and various inflammation-related conditions, offering a broader scope for developing treatment strategies (20, 21). The roles of free radicals and antioxidants in immune dysfunction and metabolic disorders have also been comprehensively studied, contributing to the potential pool of treatment options (22, 23). Furthermore, studies on the immune microenvironment emphasize the critical role of transcriptomic analysis in understanding immune cell activity, particularly in complex processes such as immune evasion in cancers, which is vital for developing new immunotherapy strategies (24–26). In the field of proteomics, extensive research into protein interaction networks and their modification mechanisms has underscored their crucial importance in regulating cell signaling and function, providing new therapeutic avenues for diseases such as cancer (27, 28).

This study not only aims to enhance the monitoring of disease progression and the prevention of dementia in SAH patients but also seeks to provide valuable insights into the complex interrelationships among tumors, dementia, and cerebral hemorrhage (29, 30). By integrating multi-omics data with bioinformatics analyses-particularly focusing on transcriptomics and proteomics—this research holds significant promise for advancing precision medicine. It aspires to improve diagnostic accuracy, refine prognostic assessments, and support the implementation of individualized therapeutic strategies (31–33). Identifying novel molecular targets for therapeutic intervention could enhance patient care and inform policy decisions related to the management of SAH, dementia, and cancer, thus advancing the field (34, 35). This comprehensive approach contributes to the scientific understanding of these diseases and paves the way for innovative treatments targeting their underlying molecular mechanisms (36, 37).

## Materials and methods

### Dataset acquisition

GEO (http://www.ncbi.nlm.nih.gov/geo) is a publicly accessible database hosting numerous high-throughput sequencing and microarray datasets contributed by global research institutions (38, 39). We utilized keywords such as subarachnoid hemorrhage and vascular dementia to search for gene expression datasets related to these conditions. The inclusion criteria required two independent expression profiles to originate from the same sequencing platform with the largest possible sample sizes. Moreover, the specimens included had to be derived from human sources. Ultimately, we downloaded three microarray datasets in FPKM format from the GEO database: GSE122063, GSE13353, and GSE161870. The GSE122063 dataset, sourced from frontotemporal lobe tissue, comprised gene expression profiles of 18 patients with vascular dementia (VaD) and 22 normal controls (40), which allows for a comparison of gene expression patterns that may be associated with the pathophysiology of VaD. GSE13353 contained 11 samples from ruptured aneurysm walls and 8 samples from unruptured aneurysm walls (41), which explores the molecular mechanisms underlying aneurysm rupture. The GSE161870 dataset assessed miRNA, including 2 samples from ruptured aneurysm walls and 2 control samples from intercostal arteries, allowing us to compare miRNAs’ expression in a vascular context.

### Identification of differentially expressed genes

The R package limma (42) was employed to calculate DEGs between various groups (42). An intersection analysis was conducted on genes upregulated and downregulated in both SAH and dementia, identifying common DEGs between these conditions. Probe sets lacking corresponding gene symbols were excluded, and for probe sets with the same gene symbol, their values were averaged. For mRNA, genes with an adjusted P-value < 0.05 were considered DEGs. For miRNA, only those with an adjusted P-value < 0.05 and |log2 fold change (FC)| ≥ 0.5 were identified as differentially expressed miRNAs.

### Functional enrichment analysis

The Gene Ontology (GO) database, developed by the Gene Ontology Consortium, offers straightforward annotations of gene products concerning their functions, biological pathways, and cellular locations (43, 44). The Kyoto Encyclopedia of Genes and Genomes (KEGG) Pathway database specializes in storing gene pathway information across various species. The “clusterProfiler” package was utilized for enrichment analysis, while the “org.Hs.eg.db” package facilitated the conversion between gene symbols and IDs. An adjusted P-value of < 0.05 was considered significant.

### Machine learning

A random forest model was applied to rank the variables based on their importance within the model (45, 46). Univariate logistic regression was then utilized to analyze risk factors associated with dementia occurrence. LASSO regression analysis was conducted to screen disease-related common genes further, employing 10-fold cross-validation to prevent overfitting. Principal Component Analysis (PCA) was used to visualize the performance of the included variables in classifying outcomes.

### Construction of miRNA–mRNA interaction network

The miRWalk platform facilitates the exploration of relationships between proteins of interest, including direct binding and co-regulated pathways, enabling the prediction of miRNAs that regulate core genes (47, 48). Cytoscape (http://www.cytoscape.org) was used to visualize this network, allowing observation of the interactions between core genes and miRNAs within the network.

### ROC curve analysis

To assess the predictive performance of core genes related to the disease outcomes, time-dependent Receiver Operating Characteristic (ROC) curves were plotted (49, 50). The area under the ROC curve (AUC) was calculated using the “pROC” package in R, and the visualization of the ROC curves was done with the ggplot2 package. The ROC curve evaluated the molecular classification ability concerning outcomes, where the AUC ranges from 0.5 to 1. An AUC close to 1 indicates a better diagnostic performance. An AUC between 0.5 and 0.7 suggests low accuracy, 0.7 to 0.9 indicates moderate accuracy, and above 0.9 signifies high accuracy.

### Gene set enrichment analysis

Gene Set Enrichment Analysis (GSEA) was performed using the clusterProfiler package in R (51, 52). The species selected was “Homo sapiens,” and the reference gene set was chosen from the MSigDB Collections (http://www.gsea-msigdb.org/gsea/msigdb/collections.jsp), specifically “c2.cp.v7.2.symbols.gmt [Curated].” Pathways were considered significantly enriched if they met the criteria of a False Discovery Rate (FDR) < 0.25 and an adjusted p-value < 0.05.

### The U251 cell line

The U251 cell line was obtained from the American Type Culture Collection (ATCC, Rockville, MD, USA). These cells were maintained in DMEM (Gibco, Grand Island, NY, USA) supplemented with 10% fetal bovine serum (FBS, BI, Kibbutz Beit Haemek, Israel) at a temperature of 37°C in an atmosphere of 5% CO_2_. The DOK3 gene was introduced into U251 cells through lentiviral vectors for gain-of-function experiments. The targeting sequences utilized were sourced from Open Biosystems.

### Quantitative real-time PCR

Following the manufacturer’s instructions, total RNA was extracted using the Cell Total RNA Isolation Kit (FOREGENE, Chengdu, China). RNA was reverse transcribed into cDNA using the StarScript II First-strand cDNA Synthesis Mix (GenStar, Beijing, China). Quantitative real-time PCR (qRT-PCR) was performed using the CFX96 Touch™ Real-Time PCR Detection System (BIO-RAD, USA). The results were analyzed using the 2^−ΔΔCt method, with GAPDH mRNA as the internal control.

### Cell viability assay and clonogenicity

Cell proliferation was assessed using the CCK-8 assay. Cells were seeded in a 96-well plate at a density of 1×10^6^ cells/ml and treated with various concentrations of CVB-D (0, 15, 30, 60, 120, 240 µmol/l) for 24, 48, and 72 hours. The condition resulting in a significant decrease in cell viability was 240 µmol/l for 72 hours, which was the highest concentration and longest duration used in the experiment. Each group consisted of six replicates and was performed in three independent experiments. After treatment, wells were washed with PBS (0.01 M), and 10 µl of CCK-8 solution was added to 90 µl of serum-free medium. The cells were incubated in the dark at 37°C for 1-2 hours. Absorbance was measured at 450 nm using a microplate reader (Tecan Group, Ltd.). For the clonogenic assay, T98G and Hs683 cells were treated with different concentrations of CVB-D (0, 5, 10, 20, 40, 80 µmol/l) for 24 hours. Cells were seeded at 500 cells per well in a 6-well plate and cultured for 10 days post-treatment. Subsequently, the cells were fixed with 4% paraformaldehyde (room temperature for 10 minutes) and stained with 0.05% crystal violet solution. Statistical analysis was conducted using GraphPad Prism 8 (GraphPad Software, Inc.).

### Flow cytometry analysis of apoptosis

Cells from the four groups were collected and centrifuged at 1,000 rpm for 5 minutes, then fixed in 70% pre-cold ethanol at -20°C for 24 hours. The samples were rinsed with phosphate-buffered saline (PBS) and incubated at 37°C with 1 mg/ml RNase (Sigma) for 30 minutes. After suspension in PBS, they were fixed with 70% ethanol for a minimum of 6 hours, followed by another PBS wash. Subsequently, the samples were resuspended in 1 ml of Propidium Iodide (PI) staining solution (50 µg/ml PI, Sigma), incubated in the dark at 4°C for 30 minutes, and analyzed by fluorescence-activated cell sorting (FACS) using a flow cytometer (FACScan, Becton Dickinson).

### Immunofluorescence

GBM cells were fixed with 4% paraformaldehyde for 30 minutes at room temperature. The cells were then permeabilized with 0.3% Triton X-100 in PBS for 15 minutes and blocked with normal goat serum for 1 hour at room temperature. After blocking, the cells were incubated overnight at 4°C with rabbit anti-NMDAR2B antibody (Cell Signaling, 1:300). The following day, the cells were washed and incubated with goat anti-rabbit Alexa Fluor 555-conjugated secondary antibody (Thermo Scientific, 1:500) for 1 hour at room temperature. The cell nuclei were stained with Hoechst 33258 (MO, USA). Images were captured using a confocal laser scanning microscope (Zeiss LSM710, Germany).

### LDH release assay

The release of lactate dehydrogenase (LDH) from neurons was measured using a commercial LDH assay kit. LDH activity was determined using an LDH cytotoxicity detection kit (Nanjing, China), based on a coupled enzyme reaction that converts tetrazolium salt to formazan. Absorbance was measured at 450 nm, and results were expressed as a percentage of LDH release relative to the control group. All experiments were conducted in triplicate.

### Assessment of oxidative stress and inflammatory cytokines

Indicators of oxidative stress, including superoxide dismutase (SOD), glutathione peroxidase (GSH-Px), reactive oxygen species (ROS), and malondialdehyde (MDA), were measured using commercial assay kits according to the manufacturer’s instructions. Samples were analyzed with a SpectraMax M2 spectrophotometer (Molecular Devices). SOD activity was expressed as U/mg protein (450 nm), GSH-Px activity as U/mg protein (412 nm), ROS levels as pmol/mg protein/min (excitation wavelength = 488 nm, emission wavelength = 520 nm), and MDA levels as nmol/mg protein (523 nm). Inflammatory cytokines, including TNF-α, IL-1β, and IL-6, were measured using ELISA kits after 24 hours of treatment, following the manufacturer’s guidelines. Data were analyzed using the SpectraMax M2 spectrophotometer (Molecular Devices).

### Statistical analysis

Statistical analyses for this study were performed using R software (version 4.0.2, https://www.r-project.org/). Correlation analysis was conducted using Pearson’s method. Categorical data were analyzed with the chi-square test, while continuous data were assessed using either the t-test or the Wilcoxon test. Sankey diagrams were created using the ggalluvial package [version 0.12.3]. A P-value of less than 0.05 was deemed statistically significant for all analyses.

## Results

### Machine learning-driven analysis of gene expression in SAH and dementia

**Figure 1:**
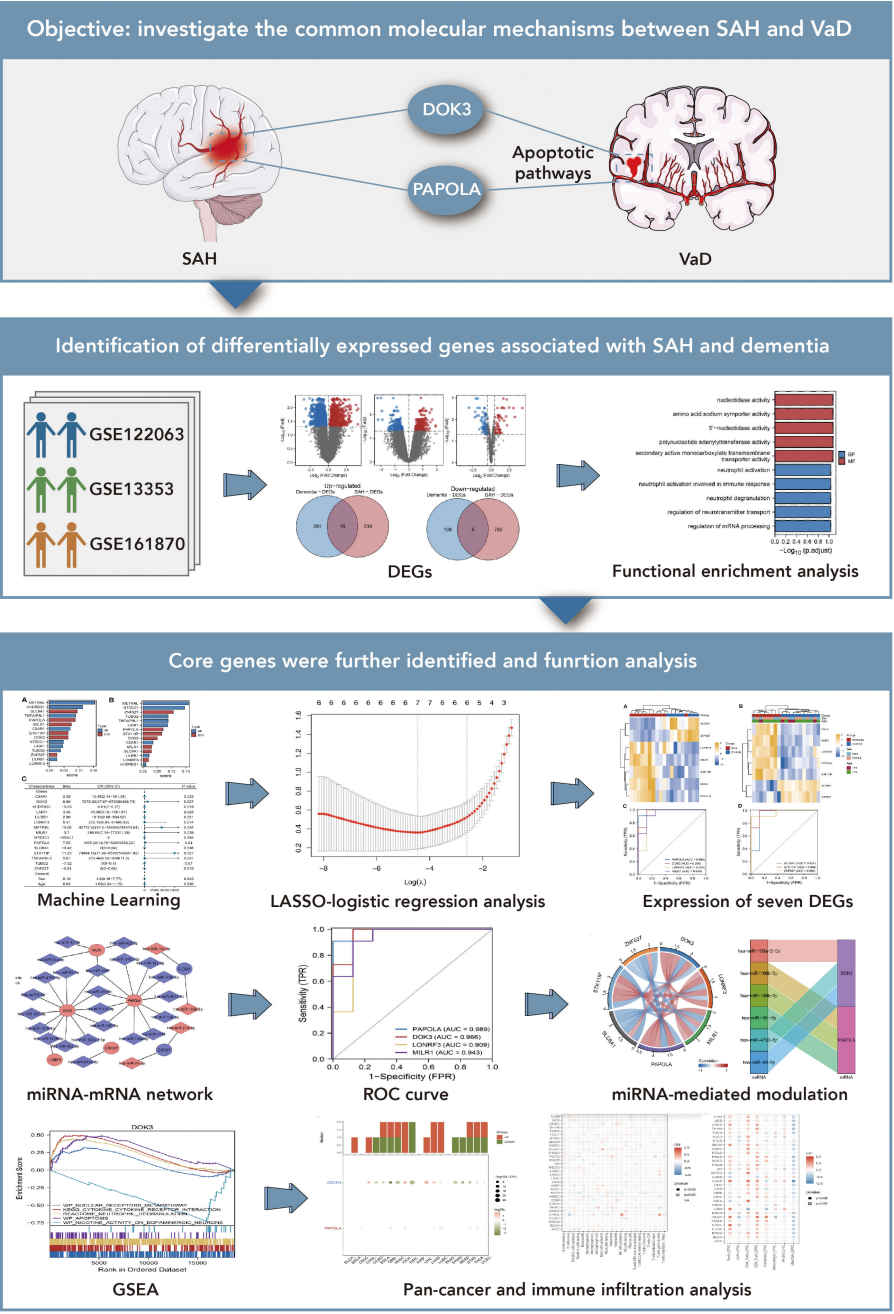
Exploration of shared molecular mechanisms between subarachnoid hemorrhage (SAH) and vascular dementia (VaD). This figure investigates the potential molecular connections between SAH and VaD, focusing on the roles of DOK3 and PAPOLA in apoptotic pathways. The upper section presents the research objective, emphasizing how these genes may mediate shared pathways in the progression of both conditions. The middle section identifies differentially expressed genes (DEGs) associated with SAH and dementia from three GEO datasets (GSE122063, GSE13353, and GSE161870). Comparative analyses, including volcano plots and Venn diagrams, were used to determine overlapping DEGs, followed by functional enrichment analysis to categorize these genes into relevant biological pathways, particularly those related to apoptosis and neurodegenerative processes. The lower section further investigates core genes using various approaches: machine learning and LASSO-logistic regression were applied to prioritize and select key genes for further analysis. Heatmaps illustrate the expression of seven significant DEGs across different conditions. Additionally, a miRNA-mRNA interaction network was constructed to explore regulatory mechanisms, and ROC curve analysis was performed to evaluate the diagnostic potential of these genes. The figure also includes a representation of miRNA-mediated regulation through circular plots. Finally, gene set enrichment analysis (GSEA) was conducted to explore the involvement of core genes in broader biological pathways, while pan-cancer and immune infiltration analyses provided insights into their expression across different cancer types and their association with immune cell infiltration.

**Figure 2:**
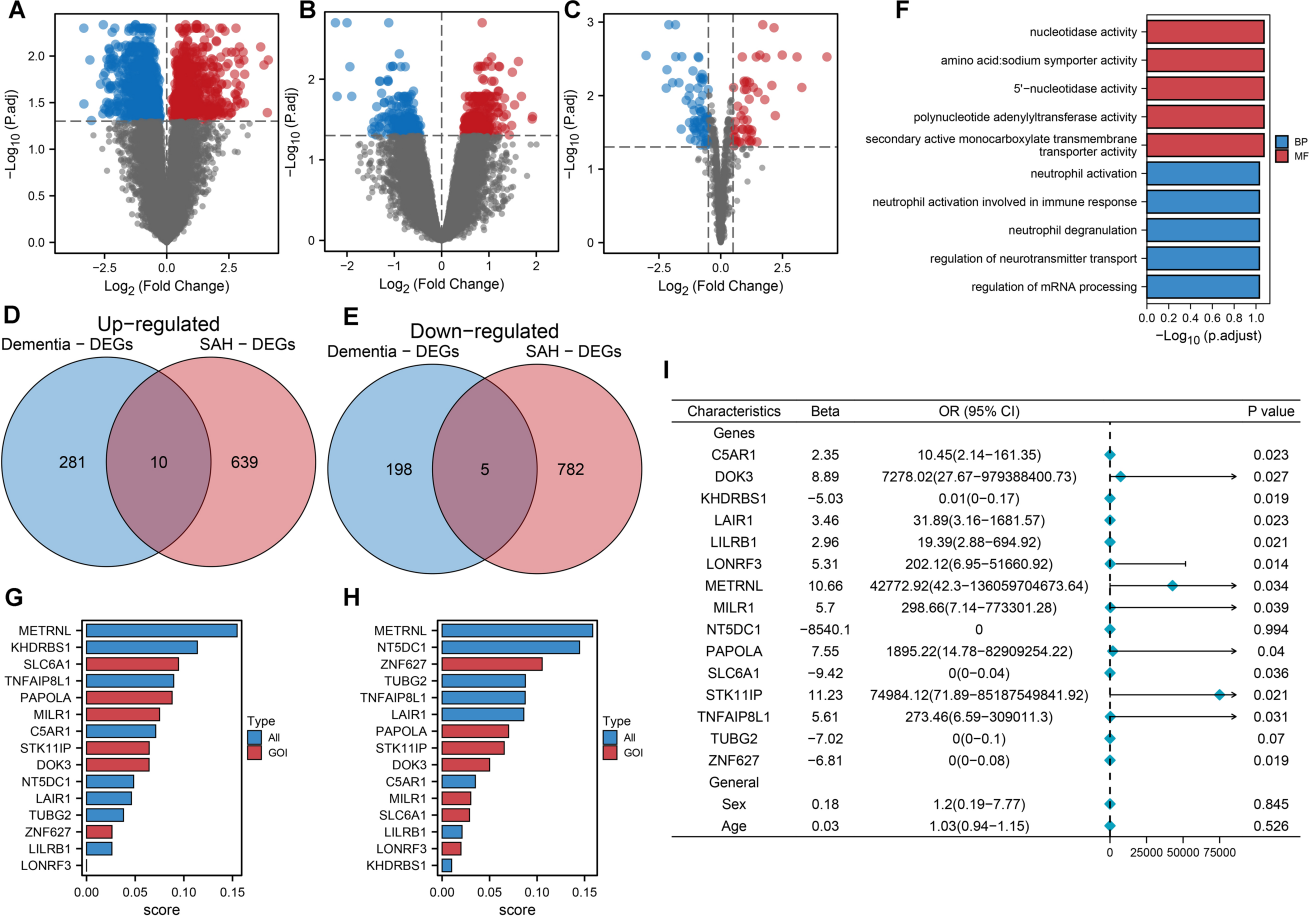
Machine learning-driven analysis of gene expression in SAH and dementia. **(A)** The Volcano plot of differentially expressed mRNAs in SAH highlights significant changes. Each point represented an mRNA, with red indicating upregulation and blue indicating downregulation, based on log2 fold change and -log10 (p-value). **(B)** Volcano plot of differentially expressed mRNAs in dementia, with similar color coding for expression changes. **(C)** Volcano plot for differentially expressed miRNAs in SAH, illustrating expression patterns for statistical significance and fold change. **(D)** Venn diagram showing 10 commonly upregulated mRNAs between SAH and dementia, indicating potential shared pathways or mechanisms. **(E)** Venn diagram displaying 5 commonly downregulated mRNAs between SAH and dementia, suggesting common regulatory effects or targets. **(F)** Functional enrichment analysis of 15 commonly differentially expressed genes between SAH and dementia, highlighting key GO and KEGG pathways, such as nucleotidase activity and neurotransmitter transport regulation. Bars represented log10 (p.adjust), categorizing pathways into biological process (BP) and molecular function (MF). **(G)** Random forest model ranking of 15 common differentially expressed genes for classifying SAH (subarachnoid hemorrhage) from normal controls. The top five most essential genes were METRNL, KHDRBS1, SLC6A1, TNFAIP8L1, and PAPOLA, indicating their significant role in disease classification. **(H)** Random forest model ranking for classifying dementia from normal controls, highlighting METRNL, NT5DC1, ZNF627, TUBG2, and TNFAIP8L1 as the top five genes, emphasizing their importance in distinguishing dementia cases. **(I)** Univariate logistic regression analysis of the 15 shared differentially expressed genes, along with sex and age, to predict dementia. Thirteen genes showed significant statistical association with dementia occurrence, with odds ratios (OR) and 95% confidence intervals (CI) displayed. This analysis underscored the predictive value of these genes in dementia.

**Figure 1** explores the shared molecular mechanisms between subarachnoid hemorrhage (SAH) and vascular dementia (VaD), focusing on the roles of DOK3 and PAPOLA in apoptotic pathways. Using datasets from GSE122063, GSE13353, and GSE161870, differentially expressed genes (DEGs) were identified through comparative analyses, including volcano plots and Venn diagrams. Functional enrichment analysis revealed their involvement in apoptosis and neurodegenerative processes. Core genes were prioritized through LASSO-logistic regression and machine learning, with heatmaps depicting their expression profiles. miRNA-mRNA interaction networks and circular plots illustrated regulatory mechanisms, while gene set enrichment analysis (GSEA) and pan-cancer analyses provided insights into broader pathways and immune infiltration. In this study, we utilized machine learning techniques to identify 15 common DEGs between SAH and dementia, discovering 494 DEGs in GSE122063, 1436 in GSE13353, and 115 miRNAs in GSE161870 (**Figures 2A–C**). Venn diagram analysis (**Figures 2D, E**) revealed 10 upregulated and 5 downregulated DEGs shared between the conditions, including C5AR1, DOK3, KHDRBS1, etc. Functional enrichment analysis (**Figure 2F**) revealed vital pathways such as mRNA processing regulation, neurotransmitter transport control, and neutrophil degranulation, indicating significant roles of these genes in nucleotide activity, transporter activity, and immune response modulation. The random forest model identified key DEGs crucial for classifying SAH and dementia, with METRNL, KHDRBS1, SLC6A1, TNFAIP8L1, and PAPOLA being the primary discriminators for SAH, and METRNL, NT5DC1, ZNF627, TUBG2, and TNFAIP8L1 crucial for dementia classification (**Figures 2G, H**). These genes are significantly associated with dementia (**Figure 2I**) and may have important implications for disease progression and pathophysiology.

### Lasso logistic regression analysis for predictive biomarkers in dementia and core differential gene expression in SAH and dementia

**Figure 3:**
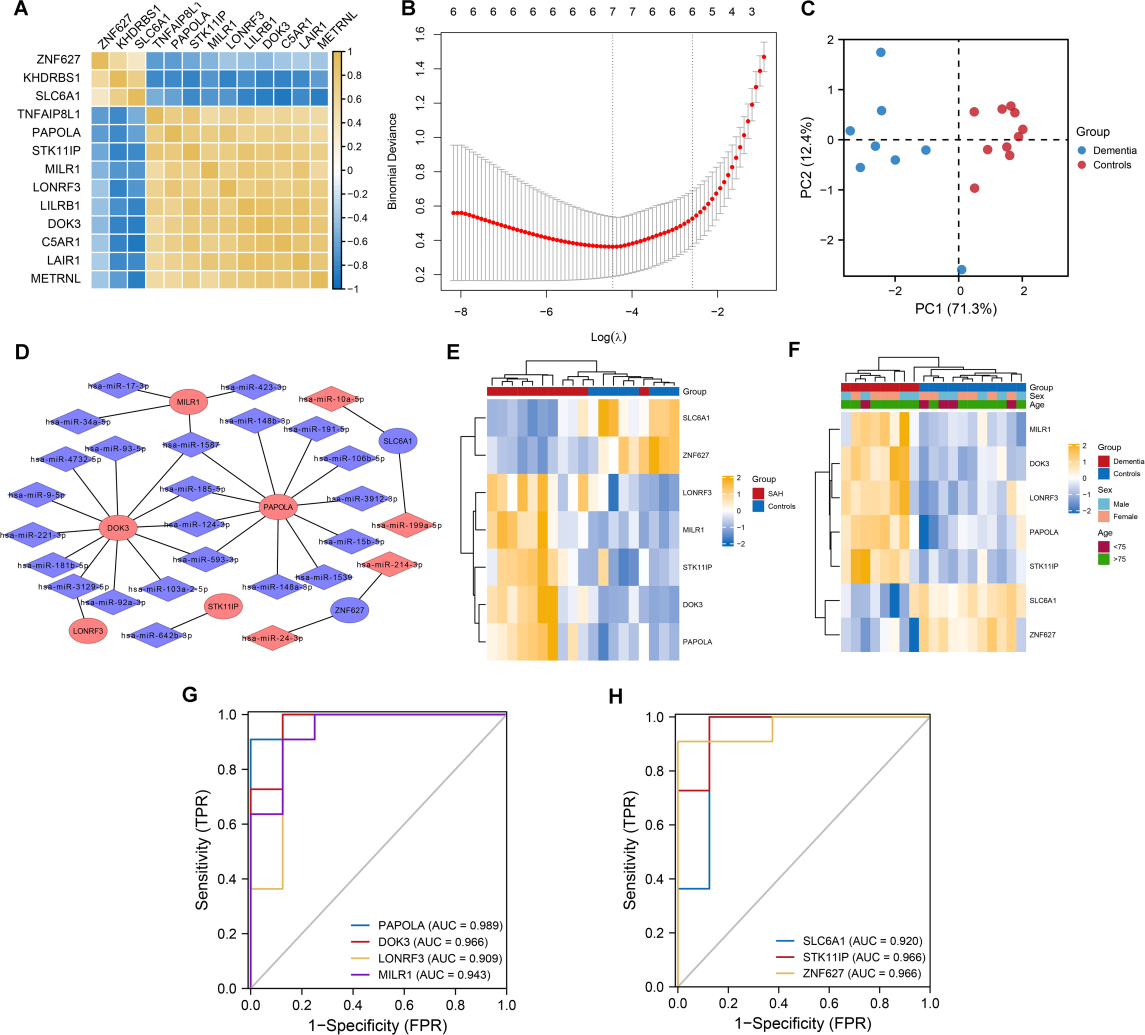
Lasso logistic regression for predictive biomarkers in dementia and core differential gene expression in SAH and dementia. **(A)** Correlation heatmap illustrating the co-expression of 13 differentially expressed genes. Notably, ZNF627, KHDRBS1, and SLC6A1 exhibited significant positive correlations, while other genes also show strong positive correlations. **(B)** Variable selection process in the LASSO-logistic regression analysis. The model identified seven genes as optimal predictors of dementia based on the minimum binomial deviance at specific lambda values. **(C)** Principal Component Analysis (PCA) plot showing a clear separation between dementia patients and normal controls based on the selected seven genes. **(D)** miRNA-mRNA interaction network depicting the interactions of differentially expressed miRNAs (DEmiRNAs) with the seven selected genes in subarachnoid hemorrhage (SAH). DOK3 and PAPOLA were highlighted as having the most connections within the network. **(E)** Heatmap illustrating the expression levels of the seven core differentially expressed genes in patients with SAH compared to controls. The color scale represented gene expression levels, with yellow indicating higher expression and blue indicating lower expression. **(F)** Heatmap showing the expression of these genes in dementia patients versus controls. Group, sex, and age were annotated above the heatmap for context. **(G, H)** Receiver Operating Characteristic (ROC) curves demonstrating the classification performance of the seven differentially expressed genes in dementia. The area under the curve (AUC) values range from 0.909 to 0.989, indicating discriminative solid ability. Each curve corresponds to a specific gene labeled with its respective AUC value.

We applied logistic regression and Lasso logistic regression analyses to identify dementia-associated genes. Univariate logistic regression analysis showed that 13 out of 15 differentially expressed genes, such as ZNF627, KHDRBS1, and SLC6A1 (**Figure 3A**), were significantly linked to dementia. Lasso logistic regression further refined this list to seven genes: DOK3, LONRF3, MILR1, PAPOLA, SLC6A1, STK11IP, and ZNF627 (**Figure 3B**). Principal Component Analysis (PCA) demonstrated a clear separation between dementia patients and controls, proving the classification effectiveness of these genes (**Figure 3C**). Additionally, we constructed a miRNA-mRNA interaction network for the seven core DEGs identified in SAH and dementia using the miRWALK website, highlighting DOK3 and PAPOLA as hub genes with the most connections (**Figure 3D**), showcasing their potential in predicting dementia and their molecular mechanisms.

### Classification and co-expression of seven differentially expressed genes in SAH: a GSEA of DOK3 and PAPOLA gene sets

**Figure 4:**
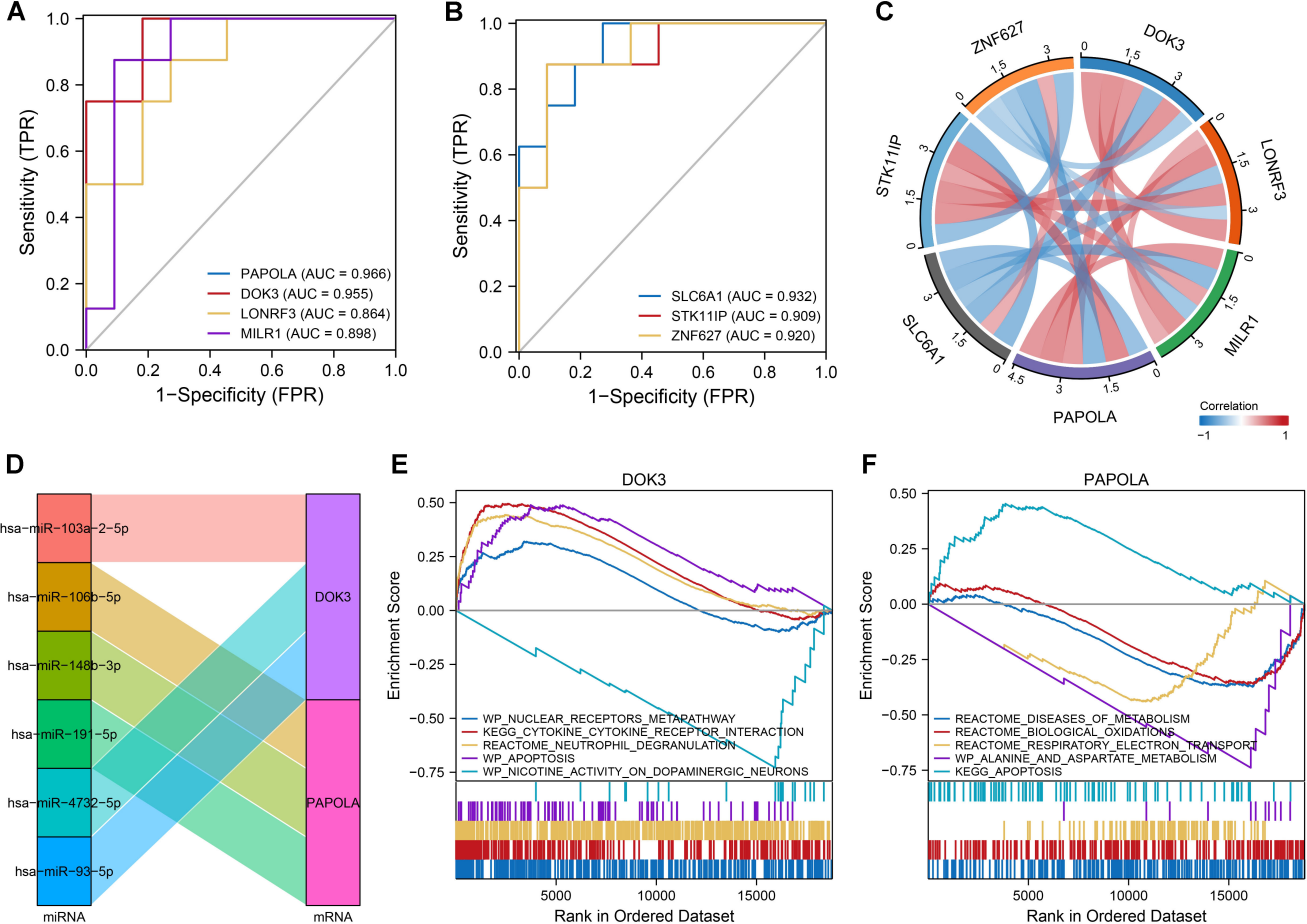
Classification and co-expression of seven differentially expressed genes in SAH. **(A, B)** ROC curves demonstrated the classification performance of the seven genes in SAH, with AUC values ranging from 0.864 to 0.966, indicating good predictive ability. Gene-specific AUC values highlighted PAPOLA, DOK3, LONRF3, MLK1, SLC6A1, STK11IP, and ZNF627. **(C)** Chord diagram illustrating the co-expression correlations among the seven genes. Notably, DOK3 and PAPOLA exhibited negative correlations with ZNF627 and SLC6A1 while showing positive correlations with the remaining genes, emphasizing the complex regulatory interactions. **(D)** Sankey diagram displaying the microRNAs (miRNAs) regulating DOK3 and PAPOLA. Each miRNA was connected to its target gene, showcasing the regulatory network and potential miRNA-mediated modulation of gene expression. **(E)** GSEA results for samples with upregulated DOK3. The plot displayed enrichment scores indicating biological pathways that were either upregulated (curves trending upwards) or downregulated (curves trending downwards). Notable pathways included the WP_NUCLEAR_RECEPTORS_METAPATHWAY, KEGG_CYTOKINE_CYTOKINE_RECEPTOR_INTERACTION, and REACTOME_NEUTROPHIL_DEGRANULATION. **(F)** GSEA results for samples with upregulated PAPOLA, highlighting significant pathways, including upregulation in apoptosis pathways. Pathways were labeled with their respective enrichment scores acrossed the ordered dataset, providing insight into the differential pathway activation associated with PAPOLA expression. Data analysis was performed using standard GSEA methodology, with specific pathways identified based on their enrichment scores and rank in the ordered dataset.

The classification performance of seven core DEGs identified in SAH and dementia was evaluated, with ROC curve analysis demonstrating their strong discriminative capabilities, particularly PAPOLA in dementia with an AUC of up to 0.989, followed by DOK3, LONRF3, and MILR1, all exceeding 0.900 (**Figures 3G, H**). In SAH, these genes also showed good classification capabilities, with AUC values ranging from 0.864 to 0.966, highlighting PAPOLA, DOK3, and SLC6A1 as significant predictors (**Figures 4A, B**). We also explored the regulation of DOK3 and PAPOLA by specific miRNAs, finding DOK3 negatively correlated with SLC6A1 and ZNF627 but positively with LONRF3, MILR1, PAPOLA, and STK11IP; PAPOLA showed negative correlations with ZNF627 and SLC6A1 but positive with LONRF3, MILR1, STK11IP, and DOK3 (**Figures 4C, D**). Additionally, GSEA indicated that upregulation of DOK3 and PAPOLA affected crucial biological pathways potentially linked to processes like immune response modulation and apoptosis, with implications for conditions such as dementia (**Figures 4E, F**).

### Comprehensive pan-cancer and immune infiltration analysis of core genes DOK3 and PAPOLA

**Figure 5:**
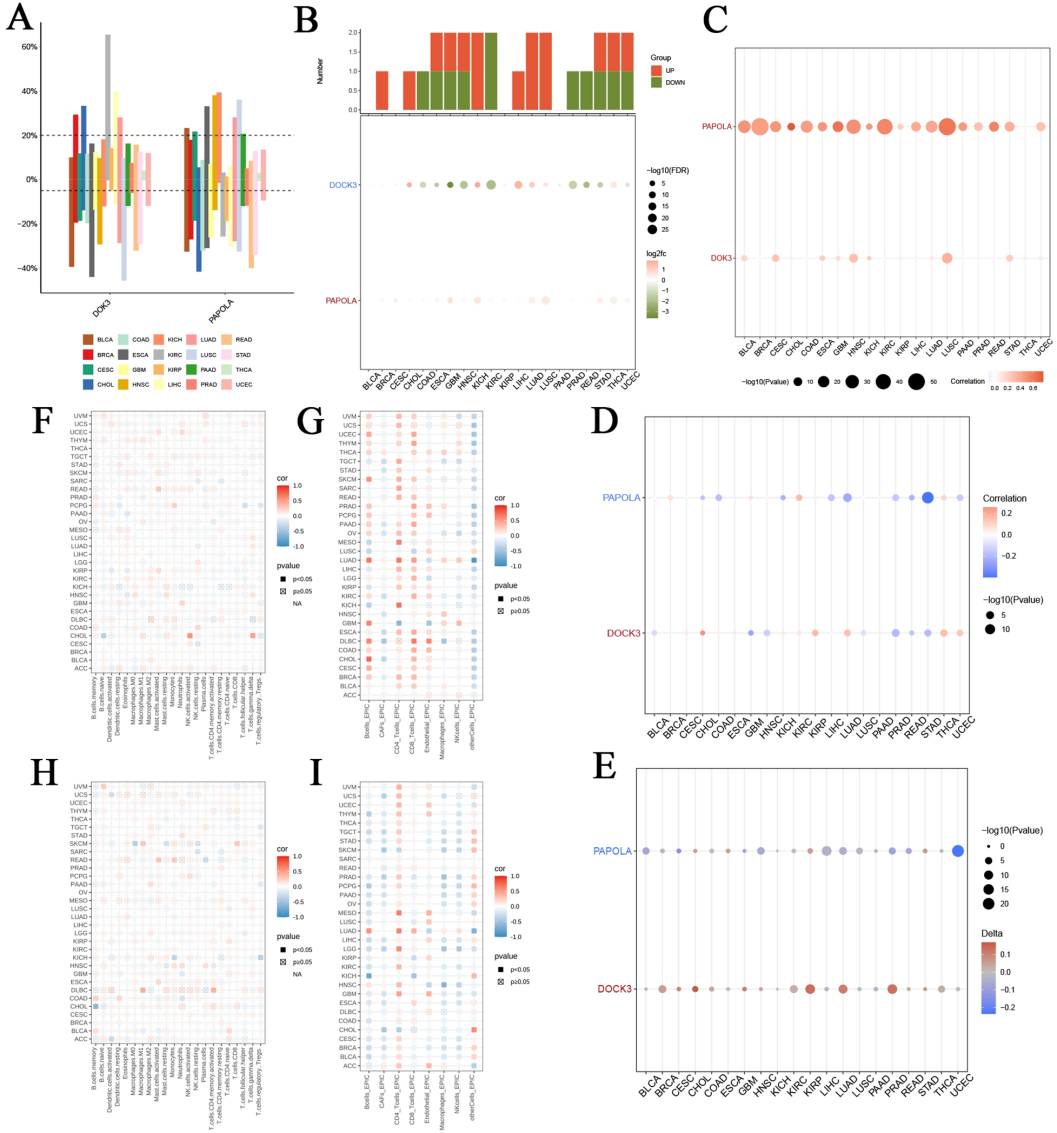
Comprehensive immune infiltration analysis of DOK3 and PAPOLA across multiple cancer types. **(A)** Distribution of copy number variation (CNV) rates of core genes (DOK3 and PAPOLA) across 20 different cancer types. Each bar represented the percentage of samples with CNV alterations in specific cancer types, color-coded by cancer type. **(B)** Differential expression of DOK3 and PAPOLA acrossed multiple cancers, showing the number of upregulated (red) and downregulated (green) samples. The lower panel showed the corresponding log2 fold change and -log10 FDR values, highlighting significant expression changes. **(C)** Correlation between CNV and expression levels of DOK3 and PAPOLA in various cancers. The size and color of the dots represented the strength and direction of the correlation, with larger and more colored dots indicating stronger correlations. **(D)** Correlation between promoter methylation levels and core gene expression (DOK3 and PAPOLA) acrossed different cancer types. The color gradient represented the correlation coefficient, with blue indicating negative, red indicating positive correlations, and dot size indicating statistical significance (-log10 p-value). **(E)** Association between tumor mutational burden (TMB) and the expression of DOK3 and PAPOLA in different cancer types. The size of the dots represented the significance level, while the color gradient showed the direction and strength of the correlation. **(F)** Immune infiltration analysis of DOK3 across cancer types, using various immune cell markers. The heatmap depicted the correlation between DOK3 expression and immune cell infiltration levels, with the color scale representing the correlation coefficient (red for positive and blue for negative). Filled squares indicated significant correlations (p<0.05). **(G)** Immune infiltration analysis of DOK3 using the EPIC method across different cancers. This heatmap displayed the correlation between DOK3 expression and immune cell infiltration levels, with the same color coding and significance representation as panel **(F, H)** The immune infiltration analysis of PAPOLA across different cancer types using various immune markers showed the correlation between PAPOLA expression and immune cell infiltration levels. The color coding and significance levels were identical to panel **(F, I)** Immune infiltration analysis of PAPOLA using the EPIC method, displaying the correlation between PAPOLA expression and infiltration levels of immune cells across cancer types. Similar to panel **(G)** color scale and significance were represented by filled squares and color intensity.

In our pan-cancer analysis, we examined the expression of DOK3 and PAPOLA across various cancer types. **Figure 5A** depicts the distribution of copy number variation (CNV) rates across 20 cancer types, with different colors representing each type, revealing significant CNV variations that may influence tumor progression. The differential expression analysis in **Figure 5B** highlighted the significant log2 fold change and -log10 FDR values for DOK3 and PAPOLA, emphasizing their potential as biomarkers. Further correlation analyses (**Figures 5C–E**) demonstrated how genetic and epigenetic factors impact gene expression. Additionally, the expression of DOK3 and PAPOLA showed significant positive or negative correlations with various immune cell infiltration levels (**Figures 5F–H**). These findings were reinforced by the EPIC method in **Figure 5I**, indicating that PAPOLA might play a dual role in influencing immune cell presence in tumors. The consistent findings across different methodologies for DOK3 and PAPOLA emphasize their importance in shaping the immune landscape of various cancers, suggesting that these genes could be critical targets for therapeutic interventions to modulate tumor immunity. This comprehensive analysis provides a solid foundation for future studies to elucidate the precise mechanisms through which DOK3 and PAPOLA influence immune infiltration in cancer.

### Tumor prognosis and functional analysis of DOK3 and PAPOLA in glioblastoma

**Figure 6:**
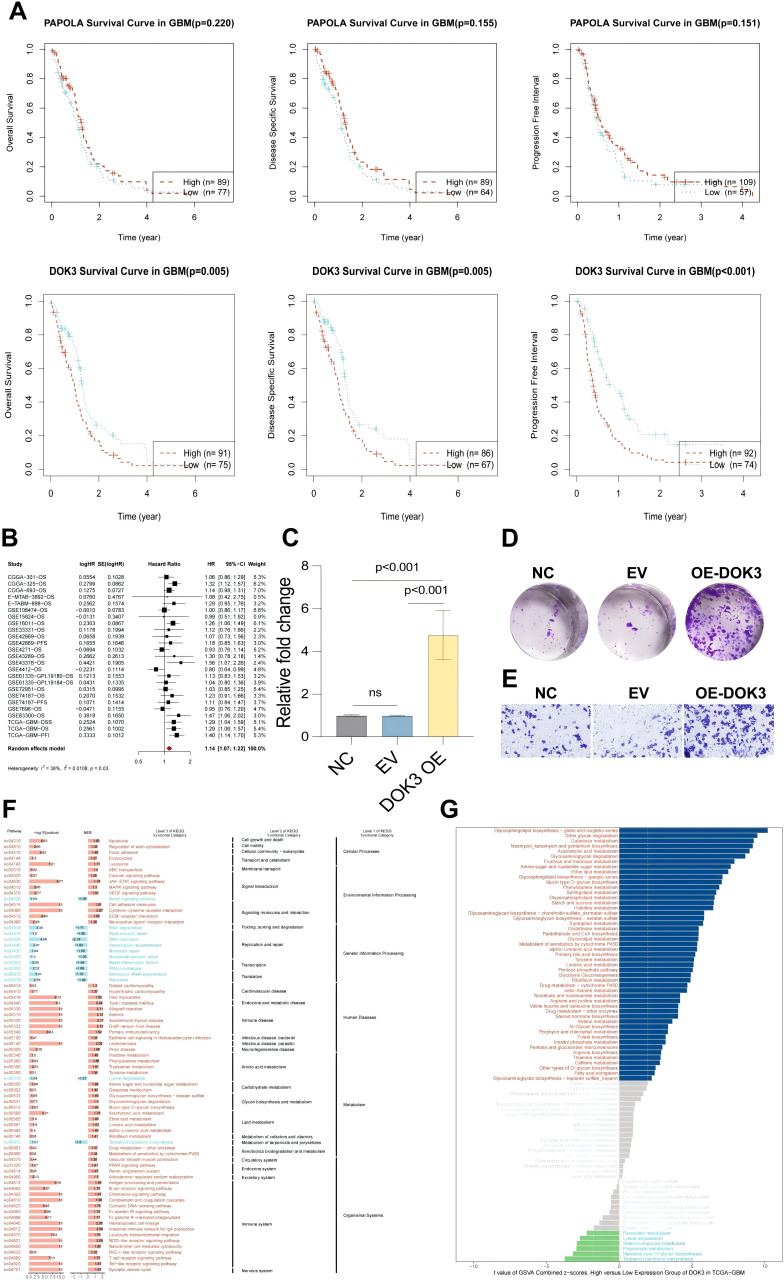
Experiments on tumor prognosis of U251MG cells. **(A)** Kaplan-Meier survival curves illustrating the association between PAPOLA and DOK3 expression and various survival outcomes in GBM patients, including overall survival (OS), disease-specific survival (DSS), and progression-free interval (PFI). The curves compared high (red) and low (blue) expression levels, with significant differences indicated in DOK3 for OS, DSS, and PFI (p-values < 0.001), while PAPOLA does not show statistically significant differences. **(B)** A meta-analysis combining hazard ratios from univariate Cox survival analysed across multiple datasets, demonstrating the prognostic value of DOK3 and PAPOLA in GBM survival. The forest plot represents the effect sizes and confidence intervals. **(C)** Bar chart showing the relative expression levels of DOK3 in cells after overexpression (OE) validation. Statistical significance was noted (p < 0.001), confirming successful overexpression compared to the control groups (NC, EV). **(D)** Colony formation assay of U251MG cells comparing the control group (NC), empty vector (EV), and DOK3 overexpression (OE-DOK3) group, with increased colony numbers in the DOK3 OE group, indicating enhanced proliferative ability. **(E)** Migration assay displaying the number of migrating cells across the membrane under different conditions (NC, EV, and OE-DOK3). The OE-DOK3 group showed a significant increase in migration ability, suggesting enhanced metastatic potential. **(F)** GSEA of KEGG pathways comparing high and low DOK3 expression groups. The heatmap highlighted critical biological processes and pathways significantly enriched in the high and low-expression groups, including metabolism, signal transduction, and disease pathways. **(G)** GSVA scores comparing metabolic pathway differences between high and low DOK3 expression groups. Metabolic pathways with the most significant differential expression were highlighted, with bars ranked based on enrichment scores.

In this study, we investigated the role of DOK3 and PAPOLA in tumor prognosis using U251MG glioblastoma cells. Kaplan-Meier survival analysis revealed that high DOK3 expression was significantly associated with poor prognosis, including worse overall survival (OS), disease-specific survival (DSS), and progression-free interval (PFI), compared to low expression (**Figure 6A**). Patients with low PAPOLA expression also demonstrated better survival rates, further suggesting the prognostic importance of these genes in glioblastoma. Meta-analysis of multiple datasets confirmed that DOK3 is a significant risk factor for glioblastoma survival (HR > 1), with moderate heterogeneity across studies (**Figure 6B**). PCR validation showed that DOK3 was successfully overexpressed in the DOK3 OE group (**Figure 6C**). Functionally, DOK3 overexpression led to increased cell proliferation, as indicated by a significant rise in colony numbers in the plate colony formation assay (**Figure 6D**), and enhanced cell migration in the Transwell migration assay (**Figure 6E**), indicating that DOK3 plays a crucial role in promoting tumor cell aggressiveness. Furthermore, GSEA and KEGG pathway analysis revealed that cell cycle-related pathways were enriched in the high DOK3 expression group, with metabolic pathways such as galactose metabolism activated and lysine degradation pathways inhibited, further suggesting that DOK3 impacts both cellular proliferation and metabolism (**Figure 6F, G**). These results collectively demonstrate the critical involvement of DOK3 and PAPOLA in glioblastoma prognosis and tumor progression, making them potential therapeutic targets for future interventions.

### Pathway and immune microenvironment analysis

**Figure 7:**
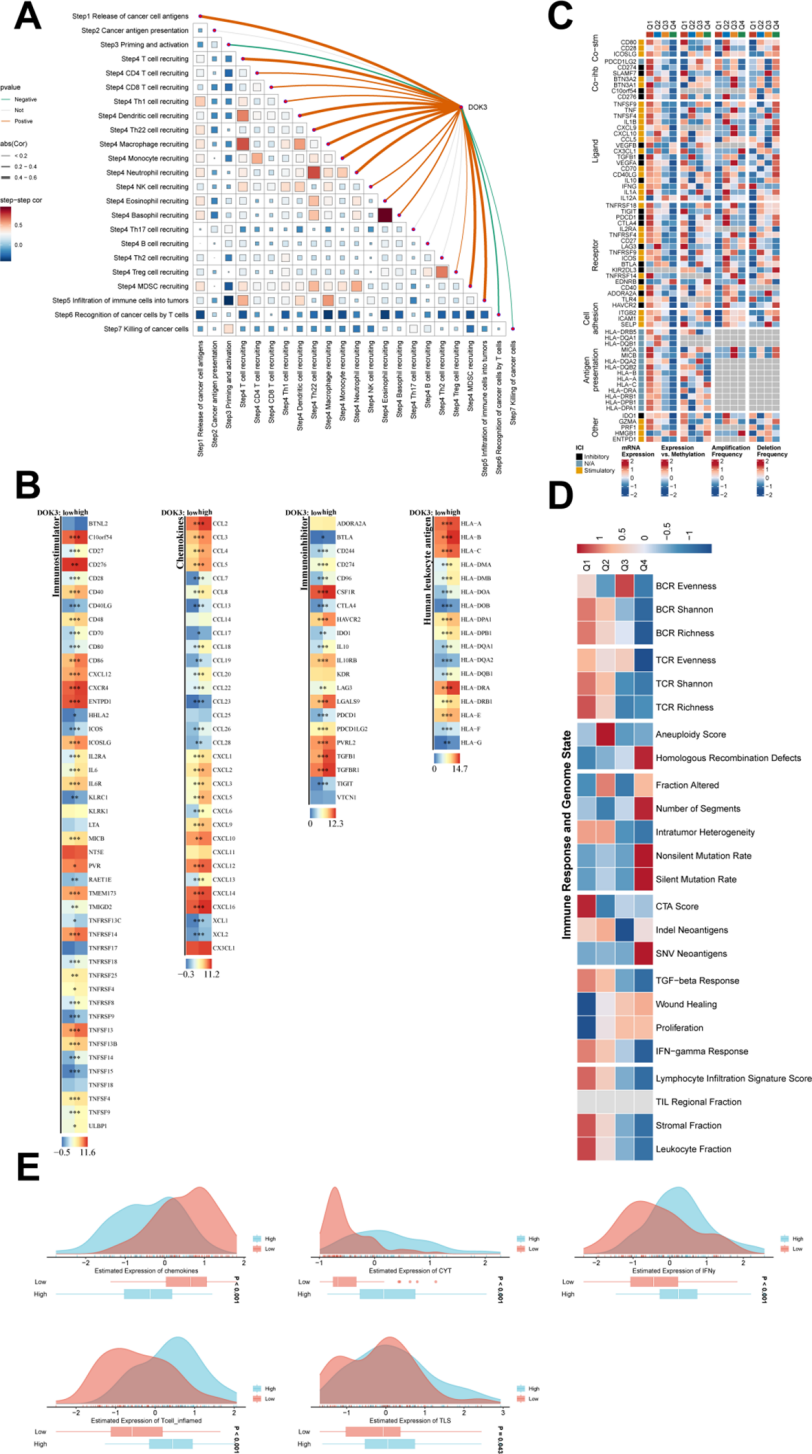
Further pathway versus immune microenvironment analysis. **(A)** The triangular heatmap illustrated the correlations between immune-related scores, focusing on the TIP (Tumor Immune Profile) scores and gene expression within the cohort. Each cell represents a correlation between two immune processes, with the intensity and direction (positive or negative) of the correlation denoted by a color gradient ranging from blue (negative correlation) to red (positive correlation). Curved lines connecting the scores highlight significant relationships, with thicker lines indicating stronger correlations. **(B)** The heatmaps depicted the expression differences in immune stimulation and suppression genes, chemokines, and HLA genes between high and low DOK3 expression groups. Each gene was color-coded, where red denotes higher expression, and blue indicated lower expression, showcasing how immune-related gene expressions vary with DOK3 levels. **(C)** A detailed heatmap illustrated the regulatory changes in various immunomodulators under different conditions or across various immune cell types. This included key factors such as antigen-presenting cells, checkpoint molecules, and other immunomodulatory proteins, indicating how these elements contributed to the immune landscape in the tumor microenvironment. **(D)** Heatmap showing the relationship between immune responses (e.g., BCR/TCR evenness and richness) and genomic instability markers (e.g., mutation rate, homologous recombination defects). Each block in the heatmap reflected the strength of association, with a color gradient from red (high association) to blue (low association). This analysis helped to elucidate how genomic characteristics could influence immune profiling and cancer progression. **(E)** Violin plots showing the distribution of MeTIL scores between high and low DOK3 expression groups. These plots visualize differences in TIL infiltration, with higher MeTIL scores typically indicative of better immune engagement in the tumor. Differences were displayed for multiple immune cell types or markers (e.g., CD8 T cells, IFN-gamma expression). The symbols "*", "**", and "***" indicate statistical significance levels: *p < 0.05, **p < 0.01, ***p < 0.001, based on comparisons between experimental and control groups in the analysis of immune and pathway-related data.

The pathway and immune microenvironment analysis revealed significant correlations between immune-related scores and gene expression levels within the cohort, as shown in **Figure 7A**. The Spearman correlation of Tumor Immune Profile (TIP) scores highlighted strong interactions between various immune processes, with thicker lines representing stronger correlations. Notably, in the DOK3 high-expression group, immune stimulation genes, immunosuppressive genes, chemokines, and human leukocyte antigen (HLA) expressions were generally elevated, as depicted in the heat maps (**Figure 7B**). These findings demonstrate that DOK3 overexpression is associated with an overall increase in immune activity. Additionally, the regulatory landscape of immunomodulators, including somatic copy number alterations (SCNA) and epigenetic factors, underscores the complex immune landscape influenced by DOK3 expression (**Figure 7C**). Further exploration of the relationship between genomic instability markers (e.g., mutation rates, homologous recombination defects) and immune responses revealed distinct patterns of immune engagement, with higher MeTIL scores observed in the high chemokine expression group. In contrast, groups with low expression of CYT, IFNγ, T cell-inflamed markers, and TLS showed lower MeTIL scores (**Figures 7D, E**). These findings underscore the role of DOK3 in modulating immune responses and its potential impact on tumor progression and immune infiltration. Integrating genomic status with immune profiles provides a comprehensive view of the tumor microenvironment and highlights potential therapeutic targets.

### Results of U251MG cells under different experimental treatments

**Figure 8:**
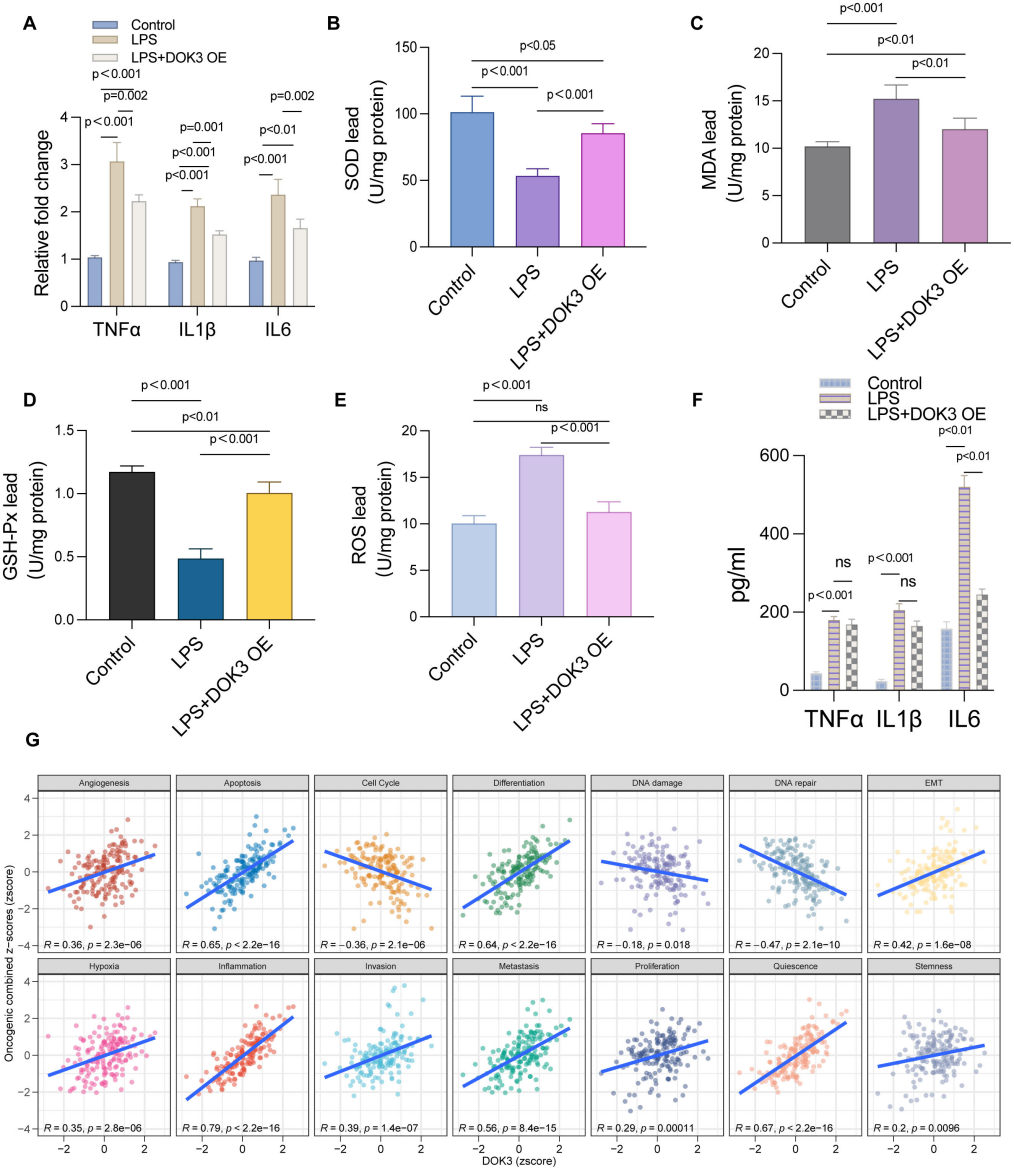
Results of U251MG cells under different experimental treatments. **(A)** The relative mRNA expression levels of key inflammatory cytokines TNF-α, IL1β, and IL-6 were measured using qPCR in U251MG cells. Three groups were compared: control, LPS (Lipopolysaccharide) treatment, and LPS with DOK3 overexpression (OE). Results showed significant upregulation of TNF-α, IL1β, and IL-6 in the LPS-treated group, which were reduced upon DOK3 overexpression (p < 0.001 for most comparisons). **(B)** A bar graph illustrating the activity of SOD in U251MG cells across different experimental groups. SOD activity was significantly reduced in the LPS-treated group compared to controls (p < 0.001) but partially restored in the LPS + DOK3 OE group (p < 0.001). **(C)** The level of MDA was measured as an indicator of lipid peroxidation in U251MG cells. MDA levels were significantly elevated in the LPS-treated group compared to the control group, with the difference being statistically significant (p < 0.001), indicating increased oxidative stress in the LPS-treated cells. As shown in **Figure 6F**, DOK3 overexpression significantly inhibited LPS-induced MDA production (p < 0.01 vs. LPS). **(D)** The activity of GSH-Px, a key enzyme in antioxidant defense, was assessed. LPS treatment resulted in a significant reduction in GSH-Px activity (p < 0.001), whereas DOK3 overexpression restored this activity to levels close to those of the control group (p < 0.001). **(E)** The levels of ROS were measured to compare oxidative stress between the experimental and control groups. LPS treatment caused a significant increase in ROS levels (p < 0.001), which was effectively reduced by DOK3 overexpression (p < 0.001), suggesting a favorable shift in oxidative balance. **(F)** Levels of cytokines (TNF-α, IL1β, IL-6) in the culture media were quantified using ELISA. The LPS group showed a significant increase in cytokine levels, which were reduced in the LPS + DOK3 OE group, indicating an anti-inflammatory effect of DOK3 overexpression (p < 0.01 for TNF-α, IL1β, and IL-6). **(G)** Pearson’s correlation analysis between DOK3 expression and tumor state scores: correlation plots showing the relationship between DOK3 z-scores and various tumor states assessed by GSVA (Gene Set Variation Analysis) z-scores. Positive correlations were observed with tumor-related processes such as angiogenesis (R = 0.36, p = 3.2e-06), apoptosis (R = 0.65, p = 2.2e-16), cell cycle (R = 0.54, p = 2.1e-06), and more, indicating DOK3’s potential involvement in these processes.

In this study, we evaluated the effects of LPS treatment and DOK3 overexpression (OE) on U251MG glioblastoma cells, focusing on inflammatory cytokine expression, oxidative stress markers, and tumor-related processes. As shown in **Figure 8A**, qPCR analysis revealed that LPS treatment significantly upregulated the expression of pro-inflammatory cytokines TNF-α, IL1β, and IL6 compared to the control group (p < 0.001). However, DOK3 OE significantly attenuated this increase (p < 0.001), indicating its potential anti-inflammatory effects. Further, **Figure 8B** demonstrates that superoxide dismutase (SOD) activity was markedly reduced following LPS treatment (p < 0.001), while DOK3 OE partially restored SOD activity to near-control levels (p < 0.001), highlighting its role in combating oxidative stress. The measurement of malondialdehyde (MDA) levels, a marker of lipid peroxidation, showed a significant increase in the LPS group (p < 0.001), as shown in **Figure 8C**, whereas DOK3 OE resulted in a dramatical reduction in MDA levels (p < 0.01). Similarly, **Figure 8D** indicates that LPS treatment significantly decreased glutathione peroxidase (GSH-Px) activity (p < 0.001), but DOK3 OE restored this activity (p < 0.001), suggesting improved antioxidant defense. The reactive oxygen species (ROS) level comparison in **Figure 8E** further confirmed that LPS treatment significantly elevated ROS levels (p < 0.001), while DOK3 OE mitigated this increase (p < 0.001), reinforcing its role in reducing oxidative damage. Additionally, **Figure 8F** illustrates the quantification of cytokines (TNF-α, IL1β, IL6) via ELISA, which revealed a significant elevation of these cytokines in the LPS group, but DOK3 OE substantially reduced their levels (p < 0.01), demonstrating an anti-inflammatory effect. Finally, **Figure 8G** shows Pearson’s correlation analysis, where DOK3 expression was positively correlated with various tumor-related processes such as apoptosis (R = 0.65, p = 2.2e-16), cell cycle (R = 0.54, p = 2.1e-06), and proliferation (R = 0.42, p = 1.6e-08), suggesting that DOK3 may play a crucial role in regulating these processes in glioblastoma cells. These results collectively indicate that DOK3 overexpression can counteract LPS-induced inflammatory and oxidative stress responses while also regulating critical tumor-related pathways.

### Anti-inflammatory and protective effects of DOK3 overexpression in BV2 cells

**Figure 9:**
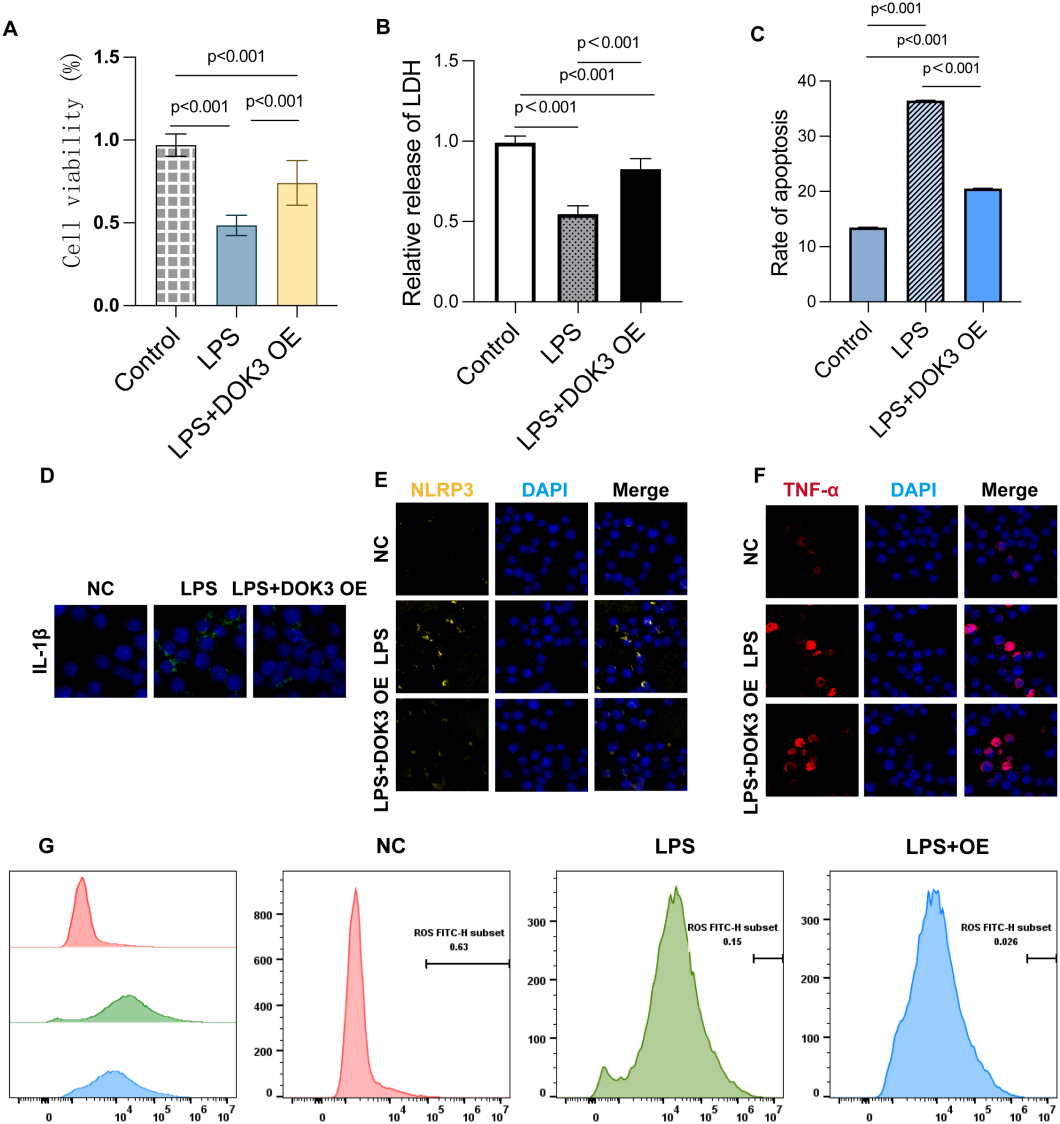
Results of BV2 cells in inflammatory studies of DOK3 anti-inflammatory effects. **(A)** The cell viability was measured by the CCK-8 assay to evaluate the proliferation and survival of BV2 cells under three conditions: control, LPS treatment, and LPS + DOK3 overexpression (OE). The results showed that LPS treatment significantly reduces cell viability (p<0.001), while DOK3 overexpression partially restores viability compared to LPS alone (p<0.001). **(B)** LDH release was measured to assess cellular damage. Cells treated with LPS exhibited significantly higher LDH release, indicating increased cytotoxicity (p < 0.001). In contrast, cells overexpressing DOK3 demonstrated reduced considerably LDH release compared to the LPS-only treatment group (p < 0.001), suggesting a protective effect of DOK3. **(C)** The extent of apoptosis was determined using flow cytometry. LPS treatment significantly increased the apoptosis rate in BV2 cells (p < 0.001), whereas DOK3 overexpression markedly decreased apoptosis compared to the LPS-only group, highlighting its anti-apoptotic effect (p < 0.001). **(D)** IL-1β expression in BV2 cells was assessed using immunofluorescence staining. LPS treatment caused a substantial increase in IL-1β expression, which was reduced in the LPS + DOK3 group, indicating DOK3’s anti-inflammatory properties. **(E)** The expression of the inflammasome marker NLRP3 was detected via immunofluorescence. LPS treatment significantly upregulated NLRP3 expression, but DOK3 overexpression reduced NLRP3 protein levels, suggesting that DOK3 may inhibit the inflammasome pathway. **(F)** LPS treatment elevated the expression of the pro-inflammatory cytokine TNF-α, whereas cells overexpressing DOK3 showed a reduction in TNF-α levels, demonstrating DOK3’s anti-inflammatory role. **(G)** ROS production was measured via flow cytometry. LPS treatment induced a significant increase in ROS production in BV2 cells, while DOK3 overexpression was associated with a reduction in ROS levels, suggesting DOK3’s protective effect against oxidative stress.

In the present study, we explored the role of DOK3 in modulating inflammation and oxidative stress in BV2 cells. LPS treatment markedly reduced cell viability, as demonstrated by the CCK-8 assay, while DOK3 overexpression (OE) partially restored cell viability, indicating its protective effect against LPS-induced cytotoxicity (**Figure 9A**, p<0.001). Furthermore, the LDH release assay showed increased LDH levels in LPS-treated cells, indicative of cell membrane damage, whereas DOK3 overexpression significantly reduced LDH release, suggesting a reduction in cellular injury (**Figure 9B**, p<0.001). Flow cytometry analysis revealed that DOK3 overexpression mitigated LPS-induced apoptosis, demonstrating its anti-apoptotic effect (**Figure 9C**, p<0.001). Immunofluorescence analysis further confirmed that DOK3 overexpression reduced the expression of key inflammatory markers, including IL-1β (**Figure 9D**), NLRP3 (**Figure 9E**), and TNF-α (**Figure 9F**), following LPS treatment. Additionally, flow cytometric analysis of ROS levels showed that LPS significantly increased ROS production, while DOK3 overexpression effectively decreased oxidative stress in BV2 cells (**Figure 9G**). Collectively, these findings indicate that DOK3 overexpression exerts significant anti-inflammatory and protective effects by reducing apoptosis, inflammation, and oxidative stress, thus highlighting its potential therapeutic applications in inflammation-related diseases and cancer research.

## Discussion

Transcriptomic techniques and bioinformatics tools are pivotal for gene function annotation and pathway enrichment analysis (53, 54). These approaches have identified numerous potential signaling pathways and regulatory networks, providing valuable evidence for understanding the mechanisms of aneurysmal subarachnoid hemorrhage (aSAH) and developing targeted therapies (55–57). This study aimed to explore the relationship between aSAH and the increased risk of dementia, emphasizing the roles of DOK3 and PAPOLA as critical genes in this process. aSAH is a devastating cerebrovascular event that leads to severe neurological deficits, coma, and even death, profoundly impacting both patients and their families (58, 59). The onset of dementia following aSAH further complicates the situation, significantly diminishing the quality of life and necessitating extensive care, which can leave the patient severely disabled (60, 61). Understanding the molecular dysfunction of DOK3 and PAPOLA in aSAH patients may provide valuable insights into the deregulated molecular pathways that contribute to dementia development, thereby suggesting novel therapeutic targets (35, 62).

This study aimed to uncover the shared molecular mechanisms between SAH and vascular dementia (VaD) (11, 63). To investigate the miRNA-mRNA regulatory network involved in aSAH-induced dementia, we focused on DOK3 and PAPOLA as central genes potentially linked to disease progression (64, 65). The involvement of these genes in apoptotic pathways suggests a functional link between their dysregulation and the development of both SAH and dementia. An initial differential analysis identified 15 common DEGs enriched in pathways related to SAH and dementia (66, 67). Further Lasso regression analysis highlighted seven core co-DEGs across both conditions: DOK3, LONRF3, MILR1, PAPOLA, SLC6A1, STK11IP, and ZNF627 (68, 69). Subsequently, a SAH-associated miRNA-mRNA regulatory network was constructed based on these core genes and SAH-related differentially expressed miRNAs (64, 70). In this network, DOK3 and PAPOLA, both upregulated, were identified as potential hub genes associated with increased dementia risk post-SAH through apoptotic pathways (68, 71).

The Dock Homolog 3 (DOK3) adapter protein, a member of the DOK protein family, plays a significant role in regulating immune responses, particularly in macrophages and B cells (72, 73). It is involved in various biological processes, such as cell migration and signal transduction. Recent genomic studies have identified DOK3 as a potential risk locus for neurological disorders, likely due to its influence on microglial activation, a critical component of the central nervous system’s innate immune response (72, 74). Emerging evidence suggests that after an aSAH, DOK3 may contribute to cognitive decline or dementia by influencing apoptotic pathways and modulating immune cell trafficking (75, 76). Moreover, DOK3 has been shown to regulate signaling cascades involved in cell proliferation, survival, invasion, and tumor microenvironment regulation, establishing its role in cancer development and progression. These findings indicate that DOK3 could be a pivotal factor in this pathway, and understanding its role may lead to the development of novel therapeutic strategies for preventing cognitive dysfunction following aSAH and in cancer.

PAPOLA is an enzyme crucial for the maturation of the 3’ end of mRNA in eukaryotic cells (77, 78). It adds a poly(A) tail to mRNA, which is essential for RNA stability, translation, and nuclear export (79, 80). Recent studies have linked PAPOLA to neurological diseases such as dementia, which may complicate the course of aSAH (81). The role of PAPOLA in post-aSAH dementia may be tied to its involvement in gene expression and mRNA stability, both critical for neuronal function and brain repair after injury (82, 83). An increase in A8RNA levels and a corresponding decrease in C9orf72 transcription, coupled with reduced PAPOLA activity, could contribute to cognitive deficits after aSAH by hindering the activation of stress response pathways (82, 84). Further research is warranted to explore PAPOLA’s impact on cognition following aSAH and to assess its potential as a therapeutic target for preventing post-aSAH dementia (85, 86).

The roles of DOK3 and PAPOLA as central players in multiple diseases, including glioma, are further supported by existing research (87, 88). Cross-disease analysis identified 15 shared DEGs between SAH and VaD, highlighting potential molecular connections. DOK3, involved in immune signaling and apoptosis regulation, and PAPOLA, crucial for mRNA stability and linked to cancer, appear to be key components of the pathways through which SAH may parallel the effects observed in VaD (84). Gene Set Enrichment Analysis (GSEA) suggested that DOK3 and PAPOLA might influence both SAH and dementia through apoptotic pathways, which aligns with literature indicating dysregulated apoptosis in neurodegenerative diseases and neurovascular injury. Excessive apoptosis, for instance, has been shown to cause neuronal loss in dementia and exacerbate brain injury post-SAH (67). The inhibition of USP30 has been found to play a significant role in modulating mitochondrial dynamics and autophagy, offering protection against early brain injury following subarachnoid hemorrhage (89). Furthermore, a pan-cancer analysis revealed that DOK3 and PAPOLA exhibit differential expression across various cancer types, reflecting their involvement in fundamental cellular processes like apoptosis and inflammation (90, 91). This cross-disease relevance highlights their potential as critical therapeutic targets (92). The dual role of these genes in both cancer and neuroinflammation broadens our understanding of their influence on oncology and neurobiology (93, 94). Targeting autophagic pathways may represent a promising and innovative approach to mitigating ischemic injury, warranting further investigation (95).

These findings align with previous research into the molecular mechanisms of SAH and dementia. Earlier studies have highlighted the distinct roles of DOK3 and PAPOLA in apoptosis and immune responses (67). However, this study introduces a novel perspective by demonstrating their cooperative effect on both SAH and VaD through bioinformatics-driven analysis (96). While prior study has often focused on individual aspects of SAH or dementia, our integrative approach provides a clearer understanding of their shared molecular pathways (66, 97). This study fills a critical gap in the literature by identifying overlapping genes and pathways, paving the way for new potential therapeutic strategies (66, 98).

DOK3 is a member of the DOK protein family, which are common substrates for multiple tyrosine protein kinases, including receptor and non-receptor tyrosine kinase signaling pathways. These pathways are involved in various cellular processes, such as proliferation, apoptosis, growth, and migration. Recent studies have identified DOK3 as a potential risk locus for neurological disorders, potentially due to its impact on microglial activation. A microarray gene expression profiling study found that DOK3 is upregulated in Alzheimer’s disease. Upstream analysis revealed that its activating molecule is LPS, and its receptor is TLR4, indicating that DOK3 inhibits the LPS signaling pathway through TLR4 receptors. Additionally, DOK3 has been shown to regulate junction proteins in tyrosine kinase signaling feedback loops and inhibit oncogenic pathways mediated by protein tyrosine kinases (PTKs), which are implicated in tumor development and progression. DOK3 has also been demonstrated to regulate multiple signaling cascades related to cell proliferation, survival, invasion, and the tumor microenvironment, establishing its importance in cancer development and progression. High DOK3 expression positively regulates pathways involved in cytokine-cytokine receptor interactions, neutrophil degranulation, and apoptosis while negatively influencing nicotine’s effects on dopaminergic neuron signaling. Similarly, elevated PAPOLA levels have been associated with activating apoptosis-related pathways and downregulating pathways involved in metabolic diseases, biooxidation, the respiratory electron transport chain, and alanine/aspartate metabolism. Studies in laryngeal cancer, SAH, and VaD indicate that these observations remain consistent across various diseases. Furthermore, integrating immune response analysis, methylation patterns, and autophagy assessments may enhance the accuracy of associations with pathogenesis and prognosis (99).

Despite the well-defined outcomes of this study, several limitations exist. A primary limitation is that the GEO datasets used contain only mRNA and miRNA expression data, which do not provide a comprehensive view of gene regulation across multiple biological levels *in vivo* (100). Moreover, variations in sample sizes across datasets may affect the statistical power and generalizability of our findings. Additionally, using bioinformatics and machine learning techniques, while powerful, may introduce biases related to data preprocessing and algorithm selection (101). The diversity of patient populations and the inherent heterogeneity in the datasets may also contribute to bias and pose challenges for data interpretation. Another limitation is the lack of *in vivo* validation of the identified central genes and pathways (102, 103). Although experimental validation provided some insights, further study using animal models or clinical samples is necessary to confirm these findings and elucidate the precise mechanisms involved (104).

Bioinformatics analysis methods, particularly gene-environment interaction analysis, have been widely applied to survival analysis of large-scale genomic data, providing insights into the molecular mechanisms underlying many complex diseases (105–107). Research should focus on validating these findings in clinical settings and exploring the therapeutic potential of targeting DOK3 and PAPOLA, particularly with patient stratification, prognosis, and precision therapies (108, 109). The role of DOK3 as a biomarker could be confirmed through prospective clinical trials or studies evaluating its predictive value for treatment outcomes. Additionally, a thorough investigation into the biological functions of DOK3, especially its role in pathways related to disease progression or therapeutic response, would be highly beneficial. Examining the functions of other identified core genes, such as LONRF3, MILR1, SLC6A1, STK11IP, and ZNF627, could also deepen our understanding of the molecular links between SAH and dementia (68). Moreover, conducting cell and animal experiments to corroborate bioinformatics results will be crucial (110, 111). In the future, integrating network pharmacology with experimental validation will offer innovative strategies for drug development (112). By modulating the function and activity of specific pathways, these drugs have demonstrated significant therapeutic effects in reducing tissue damage, offering promising new directions for treatment development (113). The application of big data and bioinformatics in disease diagnosis and prognosis has accelerated the evolution of precision medicine, promoting earlier interventions and more personalized treatment approaches (114, 115).

The integration of clinical and genomic data has led to the development of various models and tools for predicting disease progression and treatment response, significantly improving the accuracy of disease forecasts and supporting personalized medicine (116, 117). This study represents the first attempt to use a bioinformatics approach to explore the molecular mechanisms underlying dementia following SAH (118, 119). Our findings suggest that DOK3 and PAPOLA may contribute to disease progression through apoptotic pathways, acting as key players in the shared mechanisms between SAH and VaD, with significant potential for clinical application and further research (11, 12). As target molecules for therapeutic intervention in both conditions, they could serve as biomarkers for early diagnosis (120, 121). Furthermore, their involvement in apoptosis and neuroinflammation suggests that inhibiting these genes may help reduce the risk of developing SAH and dementia (122). This study provides new insights into the molecular mechanisms underlying SAH-associated cognitive decline and potential prevention or treatment strategies (66, 123). The development of novel targeted therapeutic strategies and ongoing investigations into molecular mechanisms across various diseases further deepen our understanding of SAH pathogenesis and progression, offering new perspectives and possibilities for future clinical applications (124–127).

## Conclusion

Through a comprehensive investigation of the molecular overlap between SAH and VaD, DOK3 and PAPOLA have emerged as key players in these shared mechanisms. PAPOLA and DOK3 may play a critical role in mediating neuroinflammatory response, neuronal apoptosis, and vascular dysfunction, and they participate in the post-transcriptional regulation of genes involved in neurovascular processes, such as cellular stress response. By combining bioinformatics analysis with experimental validation, we identified frequently altered pathways that could serve as platforms for targeted therapeutic interventions. These key factors have highlighted potential therapeutic targets for intervention and disease management. Targeting DOK3- or PAPOLA-related pathways could offer specific benefits for clinical practice, such as developing new treatment strategies, identifying biomarkers for early diagnosis or disease progression monitoring, and implementing personalized treatment approaches.

## Data Availability

The original contributions presented in the study are included in the article/supplementary material. Further inquiries can be directed to the corresponding author.

## Glossary

SAH: Subarachnoid Hemorrhage
VaD: Vascular Dementia
DEGs: Differentially Expressed Genes
GSEA: Gene Set Enrichment Analysis
KEGG: Kyoto Encyclopedia of Genes and Genomes
GO: Gene Ontology
PCA: Principal Component Analysis
ROC: Receiver Operating Characteristic
AUC: Area Under the Curve
FDR: False Discovery Rate
CNV: Copy Number Variation
TMB: Tumor Mutational Burden
miRNAs: MicroRNAs
lncRNA: Long Non-Coding RNA
PCG: Protein-Coding Gene
qRT-PCR: Quantitative Real-Time Polymerase Chain Reaction
DAVID: Database for Annotation, Visualization, and Integrated Discovery
TCGA: The Cancer Genome Atlas
NCBI: National Center for Biotechnology Information
ML: Machine Learning
LASSO: Least Absolute Shrinkage and Selection Operator
LR: Logistic Regression
MGE: Microarray Gene Expression
SEM: Standard Error of the Mean
GEO: Gene Expression Omnibus
HTS: High-Throughput Sequencing
FTLT: Frontotemporal Lobe Tissue
RN: Regulatory Network
miRNA-mRNA IN: miRNA-Messenger RNA Interaction Network
EPIC: Estimating the Proportion of Immune and Cancer cells

## References

[1] Onur OA Fink GR Kuramatsu JB Schwab S. Aneurysmatic subarachnoid haemorrhage. Neurol Res Pract. (2019) 1:15. doi: 10.1186/s42466-019-0015-3 33324881 PMC7650083

[2] Zhou J Guo P Guo Z Sun X Chen Y Feng H. Fluid metabolic pathways after subarachnoid hemorrhage. J Neurochemistry. (2022) 160:13–33. doi: 10.1111/jnc.15458 34160835

[3] Redekop GJ. Extracranial carotid and vertebral artery dissection: A review. Can J Neurol Sci. (2008) 35:146–52. doi: 10.1017/S0317167100008556 18574926

[4] Daou BJ Koduri S Thompson BG Chaudhary N Pandey AS. Clinical and experimental aspects of aneurysmal subarachnoid hemorrhage. CNS Neurosci Ther. (2019) 25:1096–112. doi: 10.1111/cns.13222 PMC6776745 31583833

[5] La Pira B Singh TD Rabinstein AA Lanzino G. Time trends in outcomes after aneurysmal subarachnoid hemorrhage over the past 30 years. Mayo Clinic Proc. (2018) 93:1786–93. doi: 10.1016/j.mayocp.2018.06.027 30522593

[6] Desai SV Law TJ Needham DM. Long-term complications of critical care. Crit Care Med. (2011) 39:371–9. doi: 10.1097/CCM.0b013e3181fd66e5 20959786

[7] Imam I. 700 Essential Neurology Checklists. 1st ed. New York: CRC Press (2021). doi: 10.1201/9781003221258

[8] K N. A Comprehensive Review. Dementia management and rehabilitation. GJARM. (2017) 3:45–50. doi: 10.19080/GJARM.2017.03.555609

[9] Tapu M Bicu D Tapu F Stovicek O. The efficacy of cerebrolysin in vascular dementia. J Neurological Sci. (2009) 283:286. doi: 10.1016/j.jns.2009.02.177

[10] Cipollini V Troili F Giubilei F. Vascular dementia. In: Diagnosis and Management in Dementia. Amsterdam: Elsevier (2020). p. 17–32. doi: 10.1016/B978-0-12-815854-8.00002-1

[11] Topkoru B Egemen E Solaroglu I Zhang JH. Early brain injury or vasospasm? An overview of common mechanisms. CDT. (2017) 18:245–250. doi: 10.2174/1389450117666160905112923 27593685

[12] Gao H Li J Li Q Lin Y. Identification of hub genes significantly linked to subarachnoid hemorrhage and epilepsy via bioinformatics analysis. Front Neurol. (2023) 14:1061860. doi: 10.3389/fneur.2023.1061860 36741285 PMC9893862

[13] Fu J Tang W Du P Wang G Chen W Li J. Identifying MicroRNA-mRNA regulatory network in colorectal cancer by a combination of expression profile and bioinformatics analysis. BMC Syst Biol. (2012) 6:68. doi: 10.1186/1752-0509-6-68 22703586 PMC3418553

[14] Liu B Li J Tsykin A Liu L Gaur AB Goodall GJ. Exploring complex miRNA-mRNA interactions with Bayesian networks by splitting-averaging strategy. BMC Bioinf. (2009) 10:408. doi: 10.1186/1471-2105-10-408 PMC2797807 20003267

[15] Dong N Zhang X Liu Q. Identification of therapeutic targets for Parkinson’s disease via bioinformatics analysis. Mol Med Rep. (2017) 15:731–5. doi: 10.3892/mmr.2016.6044 PMC5364854 28000864

[16] Chen Y Li H Lai L Feng Q Shen J. Identification of common differentially expressed genes and potential therapeutic targets in ulcerative colitis and rheumatoid arthritis. Front Genet. (2020) 11:572194. doi: 10.3389/fgene.2020.572194 33262784 PMC7686785

[17] Lin W-W Xu L-T Chen Y-S Go K Sun C Zhu Y-J. Single-cell transcriptomics-based study of transcriptional regulatory features in the mouse brain vasculature. BioMed Res Int. (2021) 2021:1–15. doi: 10.1155/2021/7643209 34337051 PMC8324343

[18] Chen Y Kang X Tao J Zhang Y Ying C Lin W. Reliability of synovial fluid alpha-defensin and leukocyte esterase in diagnosing periprosthetic joint infection (PJI): a systematic review and meta-analysis. J Orthop Surg Res. (2019) 14:453. doi: 10.1186/s13018-019-1395-3 31856885 PMC6921602

[19] Chen Y Sun Y Xu Y Lin W-W Luo Z Han Z. Single-cell integration analysis of heterotopic ossification and fibrocartilage developmental lineage: endoplasmic reticulum stress effector xbp1 transcriptionally regulates the notch signaling pathway to mediate fibrocartilage differentiation. Oxid Med Cell Longevity. (2021) 2021:7663366. doi: 10.1155/2021/7663366 PMC8563124 34737845

[20] Yang H Leng J Liu N Huang L. Editorial: Free radicals and antioxidants in diseases associated with immune dysfunction, inflammatory process, and aberrant metabolism. Front Endocrinol. (2024) 15:1363854. doi: 10.3389/fendo.2024.1363854 PMC10825471 38298379

[21] Cao Y Yang H Huang Y Lu J Du H Wang B. Mesenchymal stem cell-derived exosomal miR-26a induces ferroptosis, suppresses hepatic stellate cell activation, and ameliorates liver fibrosis by modulating SLC7A11. Open Med. (2024) 19:20240945. doi: 10.1515/med-2024-0945 PMC11097046 38756248

[22] Huang L Liu P Du Y Pan D Lee A Wolfe SA. A brown fat-enriched adipokine, ASRA, is a leptin receptor antagonist that stimulates appetite. (2023). doi: 10.1101/2023.09.12.557454

[23] Xu J Wang F Li Y Li P Zhang Y Xu G. Estrogen inhibits TGF−β1−stimulated cardiac fibroblast differentiation and collagen synthesis by promoting Cdc42. Mol Med Rep. (2024) 30:123. doi: 10.3892/mmr.2024.13246 38785153 PMC11130745

[24] Sanchez S Chimenti MS Lu Y Sagues E Gudino A Dier C. Modulation of the immunological milieu in acute aneurysmal subarachnoid hemorrhage: the potential role of monocytes through CXCL10 secretion. Transl Stroke Res. (2024) 15:345–357. doi: 10.1007/s12975-024-01259-4 38780865

[25] Alshammari A Pillai B Kamat P Jones TW Bosomtwi A Khan MB. Angiotensin II type 2 receptor agonism alleviates progressive post-stroke cognitive impairment in aged spontaneously hypertensive rats. Transl Stroke Res. (2024) 15:312–324. doi: 10.1007/s12975-024-01232-1 PMC12766158 38302738

[26] Shen L Jiang H. Pan-cancer and single-cell analysis of actin cytoskeleton genes related to disulfidptosis. Open Med. (2024) 19:20240929. doi: 10.1515/med-2024-0929 PMC10997004 38584831

[27] Wu T Zhang Z Huang H Wu X. RNA-seq analysis of ceRNA-related networks in the regulatory metabolic pathway of mice with diabetic nephropathy subjected to empagliflozin intervention. Archivos Españoles Urología. (2023) 76:680. doi: 10.56434/j.arch.esp.urol.20237609.83 38053423

[28] Tian Z Liu J Zeng M Zeng Q. Tong jing yi hao formula alleviates ornidazole-induced oligoasthenospermia in rats by suppressing ROS/MAPK/HIF-1 pathway. Archivos Españoles Urología. (2023) 76:596. doi: 10.56434/j.arch.esp.urol.20237608.74 37960959

[29] Springborg JB Frederiksen H-J Eskesen V Olsen NV. Trends in monitoring patients with aneurysmal subarachnoid haemorrhage. Br J Anaesthesia. (2005) 94:259–70. doi: 10.1093/bja/aei004 15516355

[30] Budohoski KP Czosnyka M Kirkpatrick PJ. The role of monitoring cerebral autoregulation after subarachnoid hemorrhage. Neurosurgery. (2015) 62:180–4. doi: 10.1227/NEU.0000000000000808 26181941

[31] Jiang L Peng Y He R Yang Q Yi C Li Y. Transcriptomic and macroscopic architectures of multimodal covariance network reveal molecular–structural–functional co-alterations. Research. (2023) 6:171. doi: 10.34133/research.0171 PMC10249784 37303601

[32] Gu Y Wang Z Wang Y Gong Y Li C. Exploring longitudinal MRI-based deep learning analysis in parkinson’s patients - A short survey focus on handedness. CI. (2024) 3:37–48. doi: 10.58567/ci03010006

[33] William C Wangmo C Ranjan A. Unravelling the application of machine learning in cancer biomarker discovery. CI. (2023) 15:123–130. doi: 10.58567/ci02010001

[34] Shah S Tereda A Lucke-Wold B Pichardo-Rojas PS. Therapeutic small molecules in the development of treatment for subarachnoid hemorrhage. ITPS. (2024) 7:2019. doi: 10.36922/itps.2019

[35] Li Y Wu P Bihl JC Shi H. Underlying mechanisms and potential therapeutic molecular targets in blood-brain barrier disruption after subarachnoid hemorrhage. CN. (2020) 18:1168–79. doi: 10.2174/1570159X18666200106154203 PMC7770641 31903882

[36] Maiti P Manna J Dunbar GL. Current understanding of the molecular mechanisms in Parkinson’s disease: Targets for potential treatments. Transl Neurodegener. (2017) 6:28. doi: 10.1186/s40035-017-0099-z 29090092 PMC5655877

[37] Redenšek S Dolžan V Kunej T. From genomics to omics landscapes of parkinson’s disease: revealing the molecular mechanisms. OMICS: A J Integr Biol. (2018) 22:1–16. doi: 10.1089/omi.2017.0181 PMC5784788 29356624

[38] Barrett T. NCBI GEO: mining millions of expression profiles–database and tools. Nucleic Acids Res. (2004) 33:D562–6. doi: 10.1093/nar/gki022 PMC539976 15608262

[39] Clough E Barrett T. The Gene Expression Omnibus Database. In: Mathé E Davis S, editors. Statistical Genomics. Methods in Molecular Biology. Springer New York, New York, NY (2016). p. 93–110. doi: 10.1007/978-1-4939-3578-9_5 PMC4944384 27008011

[40] McKay EC Beck JS Khoo SK Dykema KJ Cottingham SL Winn ME. Peri-infarct upregulation of the oxytocin receptor in vascular dementia. J Neuropathology Exp Neurol. (2019) 78:436–52. doi: 10.1093/jnen/nlz023 PMC6467199 30990880

[41] Kurki MI Häkkinen S-K Frösen J Tulamo R Von Und Zu Fraunberg M Wong G. Upregulated signaling pathways in ruptured human saccular intracranial aneurysm wall: an emerging regulative role of toll-like receptor signaling and nuclear factor-κB, hypoxia-inducible factor-1A, and ETS transcription factors. Neurosurgery. (2011) 68:1667–76. doi: 10.1227/NEU.0b013e318210f001 21336216

[42] Ritchie ME Phipson B Wu D Hu Y Law CW Shi W. limma powers differential expression analyses for RNA-sequencing and microarray studies. Nucleic Acids Res. (2015) 43:e47–7. doi: 10.1093/nar/gkv007 PMC4402510 25605792

[43] Gene Ontology Consortium. The gene ontology (GO) project in 2006. Nucleic Acids Res. (2006) 34:D322–6. doi: 10.1093/nar/gkj021 PMC1347384 16381878

[44] Thomas PD. The Gene Ontology and the Meaning of Biological Function. In: Dessimoz C Škunca N, editors. The Gene Ontology Handbook. Methods in Molecular Biology. Springer New York, New York, NY (2017). p. 15–24. doi: 10.1007/978-1-4939-3743-1_2 PMC6438694 27812932

[45] Genuer R Poggi J-M. Variable Importance. In: Random Forests with R. Springer International Publishing, Cham (2020). p. 57–76. doi: 10.1007/978-3-030-56485-8_4

[46] Behnamian A Millard K Banks SN White L Richardson M Pasher J. A Systematic approach for variable selection with random forests: achieving stable variable importance values. IEEE Geosci Remote Sens Lett. (2017) 14:1988–92. doi: 10.1109/LGRS.2017.2745049

[47] Sticht C de la Torre C Parveen A Gretz N. miRWalk: An online resource for prediction of microRNA binding sites. PloS One. (2018) 13:e0206239. doi: 10.1371/journal.pone.0206239 30335862 PMC6193719

[48] Dweep H Gretz N Sticht C. miRWalk Database for miRNA–Target Interactions. In: Alvarez ML Nourbakhsh M, editors. RNA Mapping. Methods in Molecular Biology. Springer New York, New York, NY (2014). p. 289–305. doi: 10.1007/978-1-4939-1062-5_25 25055920

[49] Zheng Y Cai T Feng Z. Application of the time-dependent ROC curves for prognostic accuracy with multiple biomarkers. Biometrics. (2006) 62:279–87. doi: 10.1111/j.1541-0420.2005.00441.x 16542256

[50] Cattaneo M Malighetti P Spinelli D. Estimating receiver operative characteristic curves for time-dependent outcomes: the stroccurve package. Stata J. (2017) 17:1015–23. doi: 10.1177/1536867X1801700415

[51] Yu G Wang L-G Han Y He Q-Y. clusterProfiler: an R package for comparing biological themes among gene clusters. OMICS: A J Integr Biol. (2012) 16:284–7. doi: 10.1089/omi.2011.0118 PMC3339379 22455463

[52] Subramanian A Tamayo P Mootha VK Mukherjee S Ebert BL Gillette MA. Gene set enrichment analysis: A knowledge-based approach for interpreting genome-wide expression profiles. Proc Natl Acad Sci USA. (2005) 102:15545–50. doi: 10.1073/pnas.0506580102 PMC1239896 16199517

[53] Thomson BR Schwendinger N Beckmann K Gentinetta T Couto D Wymann S. Haptoglobin attenuates cerebrospinal fluid hemoglobin-induced neurological deterioration in sheep. Transl Stroke Res. (2024) 15:421–33. doi: 10.1007/s12975-024-01254-9 PMC12045829 38652234

[54] Wang X Wen D Xia F Fang M Zheng J You C. Single-cell transcriptomics revealed white matter repair following subarachnoid hemorrhage. Transl Stroke Res. (2024) 15:489–501. doi: 10.1007/s12975-024-01265-6 38861152

[55] Yuan J Liao Y Zhang T Tang Y Yu P Liu Y. Integrating bulk RNA and single-cell sequencing data unveils efferocytosis patterns and ceRNA network in ischemic stroke. Transl Stroke Res. (2024) 15:402–15. doi: 10.1007/s12975-024-01255-8 38678526

[56] Xing W Zhao J Liu J Liu Z Chen G. The protective effects of sevoflurane on subarachnoid hemorrhage. Med Gas Res. (2024) 14:1–5. doi: 10.4103/2045-9912.379167 37721248 PMC10710289

[57] Cheng J Shi M Sun X Lu H. Therapeutic effect of hydrogen and its mechanisms in kidney disease treatment. Med Gas Res. (2024) 14:48–53. doi: 10.4103/2045-9912.378880 37929507 PMC10715323

[58] Svedung Wettervik T Lewén A Enblad P. Fine tuning of neurointensive care in aneurysmal subarachnoid hemorrhage: From one-size-fits-all towards individualized care. World Neurosurgery: X. (2023) 18:100160. doi: 10.1016/j.wnsx.2023.100160 36818739 PMC9932216

[59] Le Roux AA Wallace MC. Outcome and cost of aneurysmal subarachnoid hemorrhage. Neurosurg Clinics North America. (2010) 21:235–46. doi: 10.1016/j.nec.2009.10.014 20380966

[60] Rollnik JD Adner A. Neuropsychologische Langzeitfolgen und Teilhabestörungen nach aneurysmatischer Subarachnoidalblutung (aSAB). Fortschr Neurol Psychiatr. (2020) 88:33–9. doi: 10.1055/a-1003-6756 31986550

[61] Al-Khindi T Macdonald RL Schweizer TA. Cognitive and functional outcome after aneurysmal subarachnoid hemorrhage. Stroke. (2010) 41. doi: 10.1161/STROKEAHA.110.581975 20595669

[62] Richard SA. Elucidating the novel biomarker and therapeutic potentials of High-mobility group box 1 in Subarachnoid hemorrhage: A review. AIMS Neurosci. (2019) 6:316–32. doi: 10.3934/Neuroscience.2019.4.316 PMC7179354 32341986

[63] Chen J Li M Liu Z Wang Y Xiong K. Molecular mechanisms of neuronal death in brain injury after subarachnoid hemorrhage. Front Cell Neurosci. (2022) 16:1025708. doi: 10.3389/fncel.2022.1025708 36582214 PMC9793715

[64] Xu D Gareev I Beylerli O Pavlov V Le H Shi H. Integrative bioinformatics analysis of miRNA and mRNA expression profiles and identification of associated miRNA-mRNA network in intracranial aneurysms. Non-coding RNA Res. (2024) 9:471–85. doi: 10.1016/j.ncrna.2024.01.004 PMC10950608 38511055

[65] Yan Z Wu Q Cai W Xiang H Wen L Zhang A. Identifying critical genes associated with aneurysmal subarachnoid hemorrhage by weighted gene co-expression network analysis. Aging. (2021) 13:22345–60. doi: 10.18632/aging.203542 PMC8507255 34542421

[66] Ostrowski RP Colohan AR Zhang JH. Molecular mechanisms of early brain injury after subarachnoid hemorrhage. Neurological Res. (2006) 28:399–414. doi: 10.1179/016164106X115008 16759443

[67] Tian Q Liu S Han S-M Zhang W Qin X-Y Chen J-H. The mechanism and relevant mediators associated with neuronal apoptosis and potential therapeutic targets in subarachnoid hemorrhage. Neural Regeneration Res. (2022) 17:1823–1832. doi: 10.4103/1673-5374.346542 PMC9396483 35900398

[68] Ye F Liang J Wang T Wu X Li J Lan K. Bioinformatic analysis of coexpressed differentially expressed genes and potential targets for intracerebral and subarachnoid hemorrhage. World Neurosurg. (2022) 159:e442–52. doi: 10.1016/j.wneu.2021.12.070 34990842

[69] Zhao H Li S Zhu J Hua X-M Wan L. Analysis of peripheral blood cells’ Transcriptome in patients with subarachnoid hemorrhage from ruptured aneurysm reveals potential biomarkers. World Neurosurg. (2019) 129:e16–22. doi: 10.1016/j.wneu.2019.04.125 31026661

[70] Lou W Ding B Xu L Fan W. Construction of potential glioblastoma multiforme-related miRNA-mRNA regulatory network. Front Mol Neurosci. (2019) 12:66. doi: 10.3389/fnmol.2019.00066 30971889 PMC6444190

[71] Leng W Fan D Ren Z Li Q. Identification of upregulated NF-κB inhibitor alpha and IRAK3 targeting lncRNA following intracranial aneurysm rupture-induced subarachnoid hemorrhage. BMC Neurol. (2021) 21:197. doi: 10.1186/s12883-021-02156-1 33990177 PMC8120017

[72] Loh JT Teo JKH Lim H-H Lam K-P. Emerging roles of downstream of kinase 3 in cell signaling. Front Immunol. (2020) 11:566192. doi: 10.3389/fimmu.2020.566192 33133079 PMC7550416

[73] Lemay S Davidson D Latour S Veillette A. Dok-3, a novel adapter molecule involved in the negative regulation of immunoreceptor signaling. Mol Cell Biol. (2000) 20:2743–54. doi: 10.1128/MCB.20.8.2743-2754.2000 PMC85490 10733577

[74] Labzin LI Heneka MT Latz E. Innate immunity and neurodegeneration. Annu Rev Med. (2018) 69:437–49. doi: 10.1146/annurev-med-050715-104343 29106805

[75] Liu Y Wang M Hou X-O Hu L-F. Roles of microglial mitophagy in neurological disorders. Front Aging Neurosci. (2022) 14:979869. doi: 10.3389/fnagi.2022.979869 36034136 PMC9399802

[76] Bachiller S Jiménez-Ferrer I Paulus A Yang Y Swanberg M Deierborg T. Microglia in neurological diseases: A road map to brain-disease dependent-inflammatory response. Front Cell Neurosci. (2018) 12:488. doi: 10.3389/fncel.2018.00488 30618635 PMC6305407

[77] Komini C Theohari I Lambrianidou A Nakopoulou L Trangas T. PAPOLA contributes to cyclin D1 mRNA alternative polyadenylation and promotes breast cancer cell proliferation. J Cell Sci. (2021) 134:jcs252304. doi: 10.1242/jcs.252304 33712453

[78] Proudfoot NJ. Ending the message: poly(A) signals then and now. Genes Dev. (2011) 25:1770–82. doi: 10.1101/gad.17268411 PMC3175714 21896654

[79] Neve J Patel R Wang Z Louey A Furger AM. Cleavage and polyadenylation: Ending the message expands gene regulation. RNA Biol. (2017) 14:865–90. doi: 10.1080/15476286.2017.1306171 PMC5546720 28453393

[80] Zhao J Hyman L Moore C. Formation of mRNA 3′ Ends in Eukaryotes: Mechanism, Regulation, and Interrelationships with Other Steps in mRNA Synthesis. Microbiol Mol Biol Rev. (1999) 63:405–45. doi: 10.1128/MMBR.63.2.405-445.1999 PMC98971 10357856

[81] Wang W-X Springer JE Hatton KW. MicroRNAs as biomarkers for predicting complications following aneurysmal subarachnoid hemorrhage. IJMS. (2021) 22:9492. doi: 10.3390/ijms22179492 34502401 PMC8431281

[82] Yin J Li R Liu W Chen Y Zhang X Li X. RETRACTED: neuroprotective effect of protein phosphatase 2A/tristetraprolin following subarachnoid hemorrhage in rats. Front Neurosci. (2018) 12:96. doi: 10.3389/fnins.2018.00096 29535596 PMC5835096

[83] Peng J Wu Y Tian X Pang J Kuai L Cao F. High-Throughput Sequencing and Co-Expression Network Analysis of lncRNAs and mRNAs in Early Brain Injury Following Experimental Subarachnoid Haemorrhage. Sci Rep. (2017) 7:46577. doi: 10.1038/srep46577 28417961 PMC5394545

[84] Jiang Y Liu D-W Han X-Y Dong Y-N Gao J Du B. Neuroprotective effects of anti-tumor necrosis factor-alpha antibody on apoptosis following subarachnoid hemorrhage in a rat model. J Clin Neurosci. (2012) 19:866–72. doi: 10.1016/j.jocn.2011.08.038 22516550

[85] Chaudhry SR Hafez A Rezai Jahromi B Kinfe TM Lamprecht A Niemelä M. Role of damage associated molecular pattern molecules (DAMPs) in aneurysmal subarachnoid hemorrhage (aSAH). IJMS. (2018) 19:2035. doi: 10.3390/ijms19072035 30011792 PMC6073937

[86] Saugstad JA. MicroRNAs as effectors of brain function with roles in ischemia and injury, neuroprotection, and neurodegeneration. J Cereb Blood Flow Metab. (2010) 30:1564–76. doi: 10.1038/jcbfm.2010.101 PMC2932764 20606686

[87] Liu X Chen F Li W. Elevated expression of DOK3 indicates high suppressive immune cell infiltration and unfavorable prognosis of gliomas. Int Immunopharmacol. (2020) 83:106400. doi: 10.1016/j.intimp.2020.106400 32193105

[88] Guan Y Li M Qiu Z Xu J Zhang Y Hu N. Comprehensive analysis of DOK family genes expression, immune characteristics, and drug sensitivity in human tumors. J Advanced Res. (2022) 36:73–87. doi: 10.1016/j.jare.2021.06.008 PMC8799871 35127166

[89] Liu Y Yao C Sheng B Zhi S Chen X Ding P. Inhibition of USP30 promotes mitophagy by regulating ubiquitination of MFN2 by parkin to attenuate early brain injury after SAH. Transl Stroke Res. (2023) 14:238–51. doi: 10.1007/s12975-023-01228-3 38147294 PMC11976779

[90] Kim MS Chung NG Yoo NJ Lee SH. Mutational analysis of DOK2 tumor suppressor gene in acute leukemias. Leukemia Res. (2011) 35:e87–8. doi: 10.1016/j.leukres.2011.01.027 21329978

[91] Wang X Xu L Li Y Xu X Li Y. Comprehensive analysis of downstream of kinase (DOK) genes in pan-cancer. (2021). doi: 10.21203/rs.3.rs-291409/v1

[92] Bao C Tan T Wang S Gao C Lu C Yang S. A cross-disease, pleiotropy-driven approach for therapeutic target prioritization and evaluation. Cell Rep Methods. (2024) 4:100757. doi: 10.1016/j.crmeth.2024.100757 38631345 PMC11046034

[93] Thanan R Oikawa S Hiraku Y Ohnishi S Ma N Pinlaor S. Oxidative stress and its significant roles in neurodegenerative diseases and cancer. IJMS. (2014) 16:193–217. doi: 10.3390/ijms16010193 25547488 PMC4307243

[94] Morris LGT Veeriah S Chan TA. Genetic determinants at the interface of cancer and neurodegenerative disease. Oncogene. (2010) 29:3453–64. doi: 10.1038/onc.2010.127 PMC3005561 20418918

[95] Lu X Zhang J Ding Y Wu J Chen G. Novel therapeutic strategies for ischemic stroke: recent insights into autophagy. Oxid Med Cell Longevity. (2022) 2022:1–15. doi: 10.1155/2022/3450207 PMC9200548 35720192

[96] Li D Huang Z Dai Y Guo L Lin S Liu X. Bioinformatic identification of potential biomarkers and therapeutic targets in carotid atherosclerosis and vascular dementia. Front Neurol. (2023) 13:1091453. doi: 10.3389/fneur.2022.1091453 36703641 PMC9872033

[97] Raz L Knoefel J Bhaskar K. The neuropathology and cerebrovascular mechanisms of dementia. J Cereb Blood Flow Metab. (2016) 36:172–86. doi: 10.1038/jcbfm.2015.164 PMC4758551 26174330

[98] Fernández-Pérez I Macias-Gómez A Suárez-Pérez A Vallverdú-Prats M Giralt-Steinhauer E Bojtos L. The role of epigenetics in brain aneurysm and subarachnoid hemorrhage: A comprehensive review. IJMS. (2024) 25:3433. doi: 10.3390/ijms25063433 38542406 PMC10970583

[99] Kang X Chen Y Yi B Yan X Jiang C Chen B. An integrative microenvironment approach for laryngeal carcinoma: the role of immune/methylation/autophagy signatures on disease clinical prognosis and single-cell genotypes. J Cancer. (2021) 12:4148–71. doi: 10.7150/jca.58076 PMC8176413 34093817

[100] MaChado R Sachinidis A Futschik ME. Detection of Novel Potential Regulators of Stem Cell Differentiation and Cardiogenesis through Combined Genome-Wide Profiling of Protein-Coding Transcripts and microRNAs. Cells. (2021) 10:2477. doi: 10.3390/cells10092477 34572125 PMC8469649

[101] Franklin G Stephens R Piracha M Tiosano S Lehouillier F Koppel R. The sociodemographic biases in machine learning algorithms: A biomedical informatics perspective. Life. (2024) 14:652. doi: 10.3390/life14060652 38929638 PMC11204917

[102] Liu J Hua P Hui L Zhang L-L Hu Z Zhu Y-W. Identification of hub genes and pathways associated with hepatocellular carcinoma based on network strategy. Exp Ther Med. (2016) 12:2109–19. doi: 10.3892/etm.2016.3599 PMC5039750 27703495

[103] Frost HR Amos CI. A multi-omics approach for identifying important pathways and genes in human cancer. BMC Bioinf. (2018) 19:479. doi: 10.1186/s12859-018-2476-8 PMC6292115 30541428

[104] Fakhoury M. New insights into the neurobiological mechanisms of major depressive disorders. Gen Hosp Psychiatry. (2015) 37:172–7. doi: 10.1016/j.genhosppsych.2015.01.005 25772946

[105] Vasilik MP Belova NI Lazareva EM Kononenko NV Fedoreyeva LI. Salt tolerance assessment in triticum aestivum and triticum durum. Front Biosci (Landmark Ed). (2024) 29:150. doi: 10.31083/j.fbl2904150 38682196

[106] Li T Feng W Yan W Wang T. From metabolic to epigenetic: Insight into trained macrophages in atherosclerosis (Review). Mol Med Rep. (2024) 30:145. doi: 10.3892/mmr.2024.13269 38904193

[107] Wang J-H Yang C-T. Identification of gene-environment interactions by non-parametric kendall’s partial correlation with application to TCGA ultrahigh-dimensional survival genomic data. Front Biosci (Landmark Ed). (2022) 27:225. doi: 10.31083/j.fbl2708225 36042165

[108] Veeck J Dahl E. Targeting the Wnt pathway in cancer: The emerging role of Dickkopf-3. Biochim Biophys Acta (BBA) - Rev Cancer. (2012) 1825:18–28. doi: 10.1016/j.bbcan.2011.09.003 21982838

[109] Wang Y-F Hu Y-Q Hu Y-N Bai Y-C Wang H Zhang Q. Expression and clinical significance of DOK3 in renal clear cell carcinoma. J Int Med Res. (2023) 51:30006052311749. doi: 10.1177/03000605231174974 PMC10226309 37235715

[110] Xia L Yang Z Xv M Wang G Mao Y Yang Y. Bioinformatics analysis and experimental verification of TIGD1 in non-small cell lung cancer. Front Med. (2024) 11:1374260. doi: 10.3389/fmed.2024.1374260 PMC11034383 38651061

[111] Defreitas S Rowe M Paculis L Jia D. Integration of bioinformatics approaches and experimental validations to understand the role of notch signaling in ovarian cancer. JoVE. (2020), 60502. doi: 10.3791/60502 31984955

[112] Huang W-B Qin S-Y Zou J-B Li X Kang W-L Yuan P-W. Efficacy of Juanbi capsule on ameliorating knee osteoarthritis: a network pharmacology and experimental verification-based study. Tradit Med Res. (2024) 9:33. doi: 10.53388/TMR20230829002

[113] Zhou B-Y Yang J Luo R-R Sun Y-L Zhang H-T Yang A-X. Dexmedetomidine alleviates ischemia/reperfusion-associated acute kidney injury by enhancing autophagic activity via the α2-AR/AMPK/mTOR pathway. Front Biosci (Landmark Ed). (2023) 28:323. doi: 10.31083/j.fbl2812323 38179733

[114] Wu J Zhi Z Xu W Li D Li Q Han Y. LIM1863 is useful to explore collective cancer cell migration, and the group of heterogeneous cells undergoing collective migration behaves like a supracellular unit. BIOCELL. (2023) 47:2671–80. doi: 10.32604/biocell.2023.043494

[115] Ou S Xu Y Liu Q Yang T Chen W Yuan X. Analysis of large datasets for identifying molecular targets in intestinal polyps and metabolic disorders. BIOCELL. (2024) 48:415–29. doi: 10.32604/biocell.2024.046178

[116] Li M Liu X Jiang M Lei Y Li Z Li S. Prognostic capability of clinical SYNTAX score in patients with complex coronary artery disease and chronic renal insufficiency undergoing percutaneous coronary intervention. Rev Cardiovasc Med. (2024) 25:18. doi: 10.31083/j.rcm2501018 39077637 PMC11262395

[117] Figueredo VM. The heart renaissance. Rev Cardiovasc Med. (2024) 25:91. doi: 10.31083/j.rcm2503091 39076946 PMC11263855

[118] Kim BJ Youn DH Chang IB Kang K Jeon JP. Identification of differentially-methylated genes and pathways in patients with delayed cerebral ischemia following subarachnoid hemorrhage. J Korean Neurosurg Soc. (2022) 65:4–12. doi: 10.3340/jkns.2021.0035 34320780 PMC8752893

[119] Kikkawa Y. Gene expression profiling and bioinformatic analysis of rabbit basilar artery after experimental subarachnoid hemorrhage. J Neurol Neurophysiol. (2014) 5:101–7. doi: 10.4172/2155-9562.1000201

[120] Zhou S Jin J Wang J Zhang Z Freedman JH Zheng Y. miRNAS in cardiovascular diseases: potential biomarkers, therapeutic targets and challenges. Acta Pharmacol Sin. (2018) 39:1073–84. doi: 10.1038/aps.2018.30 PMC6289363 29877320

[121] Vargas-Sierra O Hernández-Juárez J Uc-Uc PY Herrera LA Domínguez-Gómez G Gariglio P. Role of SLC5A8 as a tumor suppressor in cervical cancer. Front Biosci (Landmark Ed). (2024) 29:16. doi: 10.31083/j.fbl2901016 38287802

[122] Wu Y Xu Y Sun J Dai K Wang Z Zhang J. Inhibiting RIPK1-driven neuroinflammation and neuronal apoptosis mitigates brain injury following experimental subarachnoid hemorrhage. Exp Neurol. (2024) 374:114705. doi: 10.1016/j.expneurol.2024.114705 38290652

[123] Dietrich HH Dacey RG. Molecular keys to the problems of cerebral vasospasm. Neurosurgery. (2000) 46:517–30. doi: 10.1097/00006123-200003000-00001 10719847

[124] Zhang M Yang B Ren T Wang X Chen H Lu C. Dual engine-driven bionic microneedles for early intervention and prolonged treatment of Alzheimer’s disease. J Controlled Release. (2024) 367:184–96. doi: 10.1016/j.jconrel.2024.01.030 38242212

[125] Li J Ma J Feng Q Xie E Meng Q Shu W. Building osteogenic microenvironments with a double-network composite hydrogel for bone repair. Research. (2023) 6:21. doi: 10.34133/research.0021 PMC10076009 37040486

[126] Lan Y-Y Cheng T-C Lee Y-P Wang C-Y Huang B-M. Paclitaxel induces human KOSC3 oral cancer cell apoptosis through caspase pathways. BIOCELL. (2024) 48:1047–54. doi: 10.32604/biocell.2024.050701

[127] Ali A Obireddy SR Lai W-F. Farnesol as a multifunctional candidate for treatment development. BIOCELL. (2024) 48:163–71. doi: 10.32604/biocell.2023.043839

